# High-Throughput Screening of Dipeptide Utilization Mediated by the ABC Transporter DppBCDF and Its Substrate-Binding Proteins DppA1-A5 in *Pseudomonas aeruginosa*

**DOI:** 10.1371/journal.pone.0111311

**Published:** 2014-10-22

**Authors:** Daniel Pletzer, Corinne Lafon, Yvonne Braun, Thilo Köhler, Malcolm G. P. Page, Michael Mourez, Helge Weingart

**Affiliations:** 1 Jacobs University Bremen, School of Engineering and Science, Bremen, Germany; 2 Sanofi-Aventis R&D, Infectious Diseases Therapeutic Unit, Toulouse, France; 3 Department of Microbiology and Molecular Medicine and Service of Infectious Diseases, University Hospital Geneva, Geneva, Switzerland; 4 Basilea Pharmaceutica International Ltd, Basel, Switzerland; Charité-University Medicine Berlin, Germany

## Abstract

In this study, we show that the *dppBCDF* operon of *Pseudomonas aeruginosa* PA14 encodes an ABC transporter responsible for the utilization of di/tripeptides. The substrate specificity of ABC transporters is determined by its associated substrate-binding proteins (SBPs). Whereas in *E. coli* only one protein, DppA, determines the specificity of the transporter, five orthologous SBPs, DppA1–A5 are present in *P. aeruginosa*. Multiple SBPs might broaden the substrate specificity by increasing the transporter capacity. We utilized the Biolog phenotype MicroArray technology to investigate utilization of di/tripeptides in mutants lacking either the transport machinery or all of the five SBPs. This high-throughput method enabled us to screen hundreds of dipeptides with various side-chains, and subsequently, to determine the substrate profile of the dipeptide permease. The substrate spectrum of the SBPs was elucidated by complementation of a penta mutant, deficient of all five SBPs, with plasmids carrying individual SBPs. It became apparent that some dipeptides were utilized with different affinity for each SBP. We found that DppA2 shows the highest flexibility on substrate recognition and that DppA2 and DppA4 have a higher tendency to utilize tripeptides. DppA5 was not able to complement the penta mutant under our screening conditions. Phaseolotoxin, a toxic tripeptide inhibiting the enzyme ornithine carbamoyltransferase, is also transported into *P. aeruginosa* via the DppBCDF permease. The SBP DppA1, and with much greater extend DppA3, are responsible for delivering the toxin to the permease. Our results provide a first overview of the substrate pattern of the ABC dipeptide transport machinery in *P. aeruginosa*.

## Introduction

*Pseudomonas aeruginosa* is a ubiquitous, opportunistic Gram-negative human pathogen that possesses high intrinsic drug resistance. Infections are difficult to eradicate since the bacterium rapidly acquires additional resistance mechanisms leading to multidrug or extensively drug resistance phenotypes. *P. aeruginosa* is the cause of hospital-acquired infections such as ventilator-associated pneumonia, bacteremia and urinary tract infections and may cause chronic infections in the lungs of cystic fibrosis patients [1]–[5].

Due to its metabolic versatility, *P. aeruginosa* is able to survive under harsh conditions in various environmental niches, where the uptake and metabolism of nutrients becomes crucial [1], [6]. The capacity to import and utilize small peptides as carbon and nitrogen sources is a very common cellular function in eukaryotic and prokaryotic organisms [7]–[9]. Furthermore, there are specific small peptides, which fulfill special biological functions as intracellular signals, antibiotics or toxins [6], [10], [11]. In recent years, bacterial peptide uptake mechanisms regained interest in antibiotic development. The so-called “Trojan-horse strategy” has been employed to deliver peptide-coupled antibacterial substances into cells through the bacterial peptide transport machinery [12], [13]. Additionally, peptide transporters are pharmaceutically relevant due to their ability to take up peptidomimetics and other related therapeutic substances [14].

Studies about the physiology of peptide transport systems have been performed in the 1970s [15] and specific uptake of di- and oligopeptides has been investigated in *Escherichia coli* and *Salmonella typhimurium* decades ago [10], [16]. Homologous transporters from other bacteria such as *Helicobacter pylori* [17], *Borrelia burgdorferi* [18], *Vibrio furnissi* [19] and *Lactococcus lactis* [20] have only been characterized quite recently.

Most of these specific bacterial transport systems belong to the ABC transporter family. ABC-type transporter systems typically consist of two permease domains and various ATP-binding domains that are responsible for the energy supply [15]. Translocation of molecules through the membrane is achieved by two hydrophobic transmembrane domains (TMD), usually driven by ATP hydrolysis. The direction of the translocation process can be deduced from the availability of so-called substrate-binding proteins (SBPs). While ABC transporters involved in the efflux of compounds do not possess SBPs, it is a necessary component of a functional ABC importer [15]. SBPs are located in the periplasm and undergo a conformation change upon substrate binding. Due to this change, the bound substrate will be delivered to the integral membrane complex, released and further translocated into the cytoplasm [15], [21], [22].

It appears energetically more favorable to take up peptides instead of cleaving them in the extracellular space [23]. Small peptides permeate the outer membrane of Gram-negative bacteria via non-specific porins [24]. Once in the periplasmic space, they are further processed by proteolytic degradation such as the arginine-specific aminopeptidase [25] or they are bound by SBPs, which deliver them to their specific transporter [21]. After translocation into the cytoplasm, membrane-associated peptidases hydrolyze the delivered peptides [26].

*P. aeruginosa* possesses more than 500 ORFs (about 10% of total ORFs) encoding proteins involved in the transport of nutrients and other small molecules [27]. The dipeptide permease (Dpp) has been associated with the utilization of dipeptides as well as some tripeptides [9]. The recognition of dipeptides has specific affinity to various L-amino acids depending on their side chains [16], [21], [23]. Moreover, Dpp was also shown to mediate dipeptide chemotaxis [16], [28] and haem metabolism [29], [30] in other bacteria. During haem utilization, the SBP DppA was shown to bind haem and to deliver it to the ABC transporter [31].

Analysis of the sequenced genome of *P. aeruginosa* PA14 revealed two ABC-type transporter systems involved in the uptake of peptides: (i) an oligopeptide transporter, recently described to be involved in the uptake of uridyl peptide antibiotics [32], and (ii) a hitherto uncharacterized transporter system, which shows homology to the previously described dipeptide uptake machinery from other bacteria. In this study, we aimed to investigate and characterize the substrate profile of the putative dipeptide transporter system DppBCDF (PA14_58440–PA14_58490) from *P. aeruginosa* PA14. Investigation of peptide importers had been limited to the small number of available substrates. Our approach included the Biolog phenotype MicroArray (PM) technology to investigate di- and tripeptides utilized as nitrogen source. For the first time, we show an extensive substrate profile comprising of almost 200 dipeptides and 14 tripeptides. Our analysis gives opportunity to assess the dipeptide transporter system of *P. aeruginosa* in much more detail as has been investigated before. A mutant of the dipeptide transport system DppBCDF showed reduced ability to utilize di/tripeptides as nitrogen source. Moreover, using this high-throughput approach, we unraveled the substrate profiles of five homologous SBPs, DppA1 (PA14_58350), DppA2 (PA14_58360), DppA3 (PA14_58390), DppA4 (PA14_58420), and DppA5 (PA14_70200), involved in peptide utilization via the DppBCDF transporter system. The emphasis of our analysis was on the properties of the amino acids side chains on peptide utilization to identify chemical groups which may be added to antibacterial compounds to promote their uptake by the dipeptide uptake machinery.

## Results

### Computational analysis of the PA14 dipeptide transporter system

**Figure 1:**
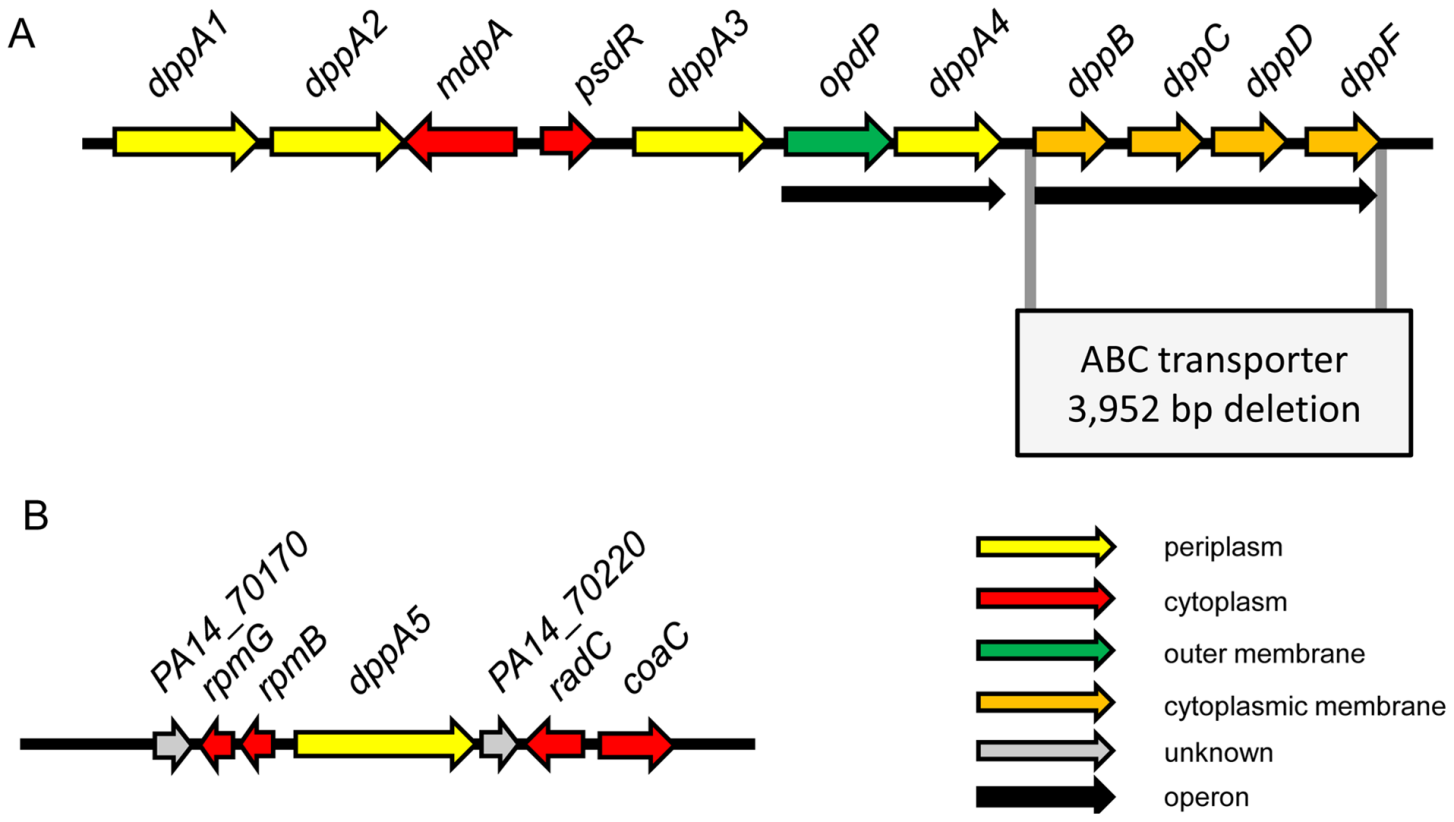
Schematic presentation of the genomic region of the *P. aeruginosa* PA14 dipeptide transport machinery. (A) Region surrounding the dipeptide transporter operon *dppBCDF* and its substrate-binding proteins *dppA1–A4*. The 3,952-bp deletion of the ABC transporter *dppBCDF* operon is indicated. (B) Genomic region surrounding *dppA5*. The subcellular location of the proteins is indicated by colors. Black arrows indicate operon structures [77].

**Figure 2:**
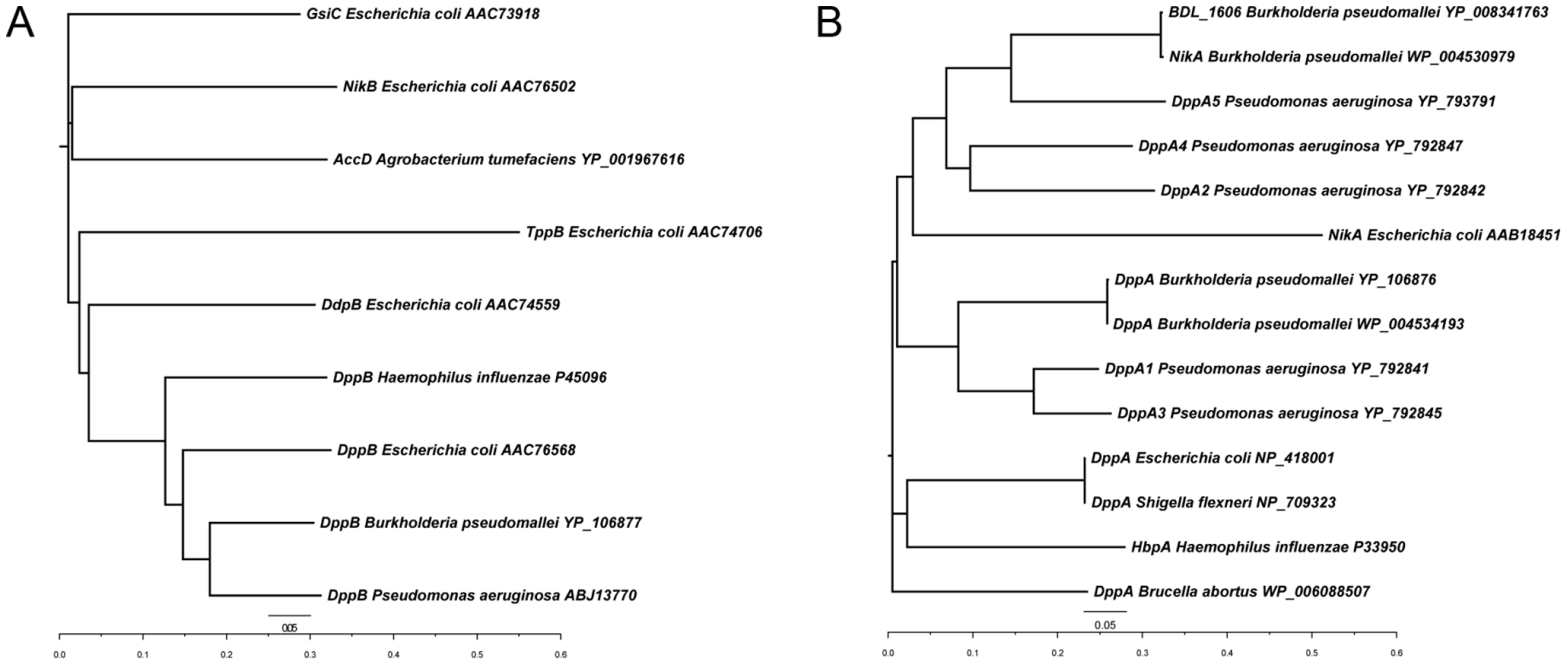
Phylogenetic relationships between (A) dipeptide transporter permeases and (B) dipeptide substrate-binding proteins.

The dipeptide ABC transporter of PA14 is encoded in an operon-like structure containing four genes, two encoding the hydrophobic permeases PA14_58440 and PA14_58450 (*dppC*), and two encoding the hydrophilic nucleotide- and ATP-binding proteins PA14_58470 (*dppD*) and PA14_58490 (*dppF*) (Figure 1A). Using the protein sequence of PA14_58440, we identified orthologs of the dipeptide permease in *Haemophilus influenzae* (61% identity), *E. coli* (66% identity) and *Burkholderia pseudomallei* (74% identity), as depicted in the phylogenetic tree in Figure 2A (see also Table S3). Since the protein sequence of PA14_58440 shows a high degree of homology to other dipeptide ABC transporters, we suggest renaming this protein as DppB and refer to the operon as the *dppBCDF* operon.

Topology analysis of the two ABC-type permeases DppB and DppC using the TOPCONS prediction software [33] revealed the typical six α-helical transmembrane spanning domains (Figure S1). Further bioinformatical analysis indicated a promoter (σ^32^) in the *dppB* upstream region [34] and a single polycistronic *dppBCDF* transcript [35].

Analysis of the genome sequence upstream of the ABC transporter (Figure 1A) revealed the presence of a putative transcriptional regulator PsdR that appears to control the *dppBCDF* operon, and MdpA, a metallo-dipeptidase contributing to cytotoxicity in PAO1 [36]. Additionally, several genes encoding periplasmic SBPs were identified: DppA1 (PA14_58350), DppA2 (PA14_58360), DppA3 (PA14_58390) and DppA4 (PA14_58420), whereas the later one appears to be co-transcribed in an operon with PA14_58410 encoding the porin OpdP (Figure 1A). Moreover, we identified a homologous SBP that is not located near the aforementioned SBPs, DppA5 (PA14_70200) (Figure 1B). Protein interaction modeling of the dipeptide transporter identified five SBPs interacting with the subunits of the transporter (Figure S2).

DppA1 and DppA3 are close paralogs (83% amino acid identity) and orthologues of DppA from *E. coli*, *S. enterica* and *B. pseudomallei* (50–60%) (Figure 2B). DppA2 and DppA4 are 59% identical to each other and show less than 60% amino acid sequence identity to the other dipeptide-binding proteins of PA14. DppA5 is not located near the *dppBCDF* operon. The DppA5 protein sequence shows about 50% identity to the other dipeptide-binding proteins of PA14 and appears to be an ortholog of BDL_1606 (bacterial extracellular solute-binding) and NikA (nickel-binding) from *B. pseudomallei*. An overview of the sequence identity data can be found in Table S4. Additionally, a graphical interpretation of the homology of the five SBPs is illustrated by the multiple sequence alignment in Figure S3.

Investigation of the transcriptional profile of the dipeptide-binding proteins in PA14, using available RNA-seq data [35], revealed that the genes encoding DppA2 and DppA5 are expressed at a lower level than the genes encoding DppA1, DppA3 and DppA4 during growth at 28°C and 37°C, respectively. These data correlate with our observations from qRT-PCR analysis with PA14 grown at 37°C (data not shown).

### Dipeptides used as nitrogen source by PA14

In order to investigate the substrate profile of dipeptides utilized by PA14, we took advantage of Biolog phenotype MicroArray plates that have numerous dipeptides implemented as nitrogen sources. This phenotype microarray approach allowed an extensive screening of the diversity of dipeptides utilized by PA14. Overall, 269 dipeptides (out of 400 possible combinations) are implemented in the Biolog plates PM6 to PM8, including 6 β-peptides containing an β-amino acid, which has the amino group bonded to the β-carbon, 2 γ-peptides (both are Glu-Gly), and 12 dipeptides containing a D-form amino acid residue. Our analysis included a subset of 162 dipeptides and 13 tripeptides. Peptides that did not promote cell respiration above the threshold value of 2000 and/or showed a strong deviation from the other replicates were disqualified.

In total, PA14 was not able to use 36 dipeptides (18%) as nitrogen source. Ten of the excluded dipeptides contained a D-form amino acid residue. A full list of all excluded dipeptides is provided in the heat maps in Figure S4 and Figure S5.

**Figure 3:**
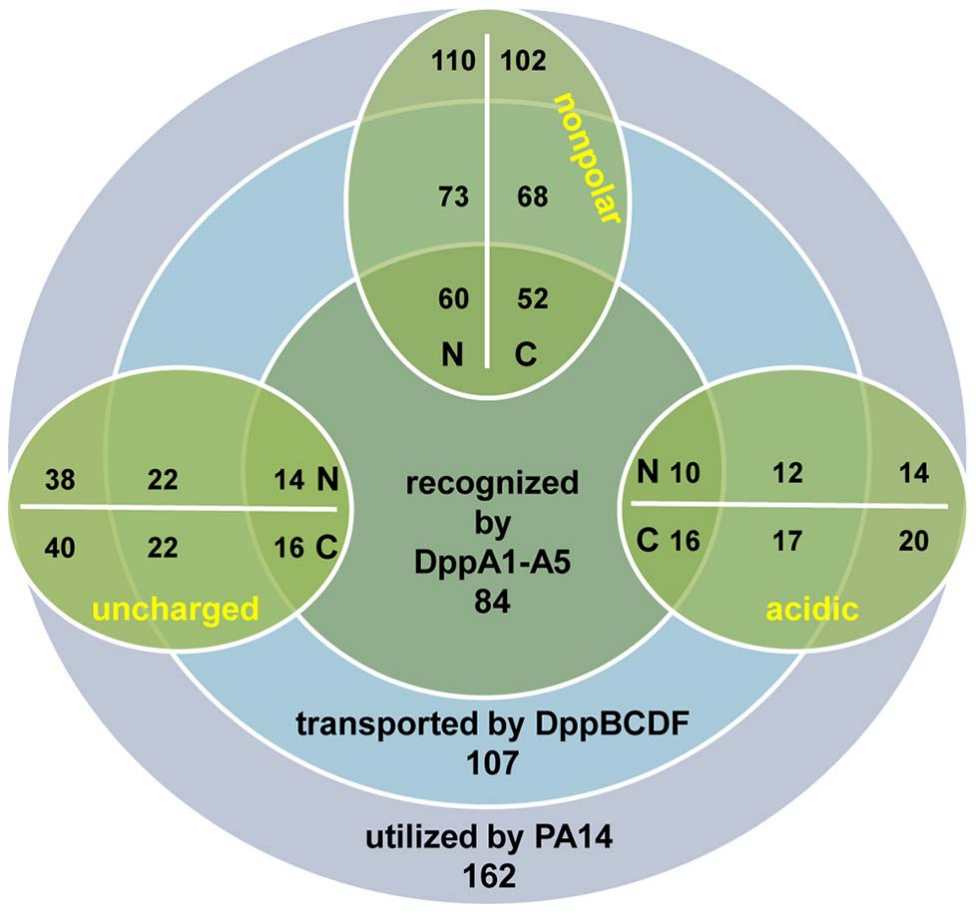
Global utilization pattern of dipeptides by PA14, the dipeptide transporter DppBCDF and the SBPs DppA1–A5. The rings show the total number of dipeptides utilized by the wild-type strain PA14 (outer purple ring), transported by the dipeptide transporter system DppBCDF (middle blue ring), and recognized by the substrate-binding proteins (inner dark green ring). The oval rings (green) present different pools of dipeptides (nonpolar, acidic, uncharged) containing a specific amino acid side-chain either at the N- or C-terminal end of the dipeptide (see Table 2). Dipeptides containing positively charged amino acid residues were excluded from the analysis (see Materials and Methods).

The remaining set of dipeptides was classified based on the properties of the side-chain group of the single amino acid residues (acidic, basic, uncharged polar or nonpolar) and the ability of PA14 to utilize them as nitrogen source (Table 1, Figure 3). Since dipeptides contain two amino acids, we clustered each dipeptide into two groups. For example, if a dipeptide contains one nonpolar and one uncharged amino acid (N- or C-terminally attached), this dipeptide was clustered into the pool of ‘nonpolar side-chains’ as well as into the pool of ‘uncharged side-chains’. This classification allowed us to identify whether specific side-chain groups are preferred by the dipeptide permease. Based on this grouping, we clustered 173 dipeptides as nonpolar, 43 as acidic, and 75 as uncharged. These clusters were then further divided based on the position of the amino acid residue in the dipeptide; either N- or C-terminal (Table 2). Basic amino acids were excluded from our analysis (see Materials and Methods).

**Table 1:** Effect of side chain properties of the terminal amino acid on utilization of dipeptides by PA14 (WT), the *dppBCDF* mutant (Dpp) and the SBP penta mutant (SBP).

Dipeptide utilization		2^nd^ amino acid (C-terminal)								
		nonpolar			acidic			uncharged		
		WT	Dpp	SBP	WT	Dpp	SBP	WT	Dpp	SBP
1^st^ amino acid (N-terminal)	nonpolar	68^a^	46	37	13	11	11	29	16	12
	acidic	12	10	8	0	0	0	2	2	2
	uncharged	22	12	7	7	6	5	9	4	2

a Numbers of dipeptides used by the strains as sole nitrogen source.

**Table 2:** Total numbers of dipeptides utilized by PA14 (WT), the *dppBCDF* mutant (Dpp) and the SBP penta mutant (SBP) (see also Figure 3).

Total	WT (162)^a^			Dpp (107)			SBP (84)		
	Nonpolar	acidic	uncharged	nonpolar	acidic	uncharged	nonpolar	acidic	uncharged
N-terminal	110	14	38	73	12	22	60	10	14
C-terminal	102	20	40	68	17	22	52	16	26
Union (N∪C)	144	34	69	95	29	40	75	26	28

a Total number of dipeptides in the pool cluster.

We found that dipeptides containing uncharged residues, either at the N- or C-terminal end, were better substrates for PA14 (69 out of 75), than nonpolar (144 out of 173) and acidic ones (34 out of 43). Dipeptides with specific side-chains were used by PA14 with approximately the same efficiency irrespective of whether the specific side-chain was at the N- or C-terminal end of the dipeptide.

### Substrate specificity of the ABC transporter DppBCDF

**Figure 4:**
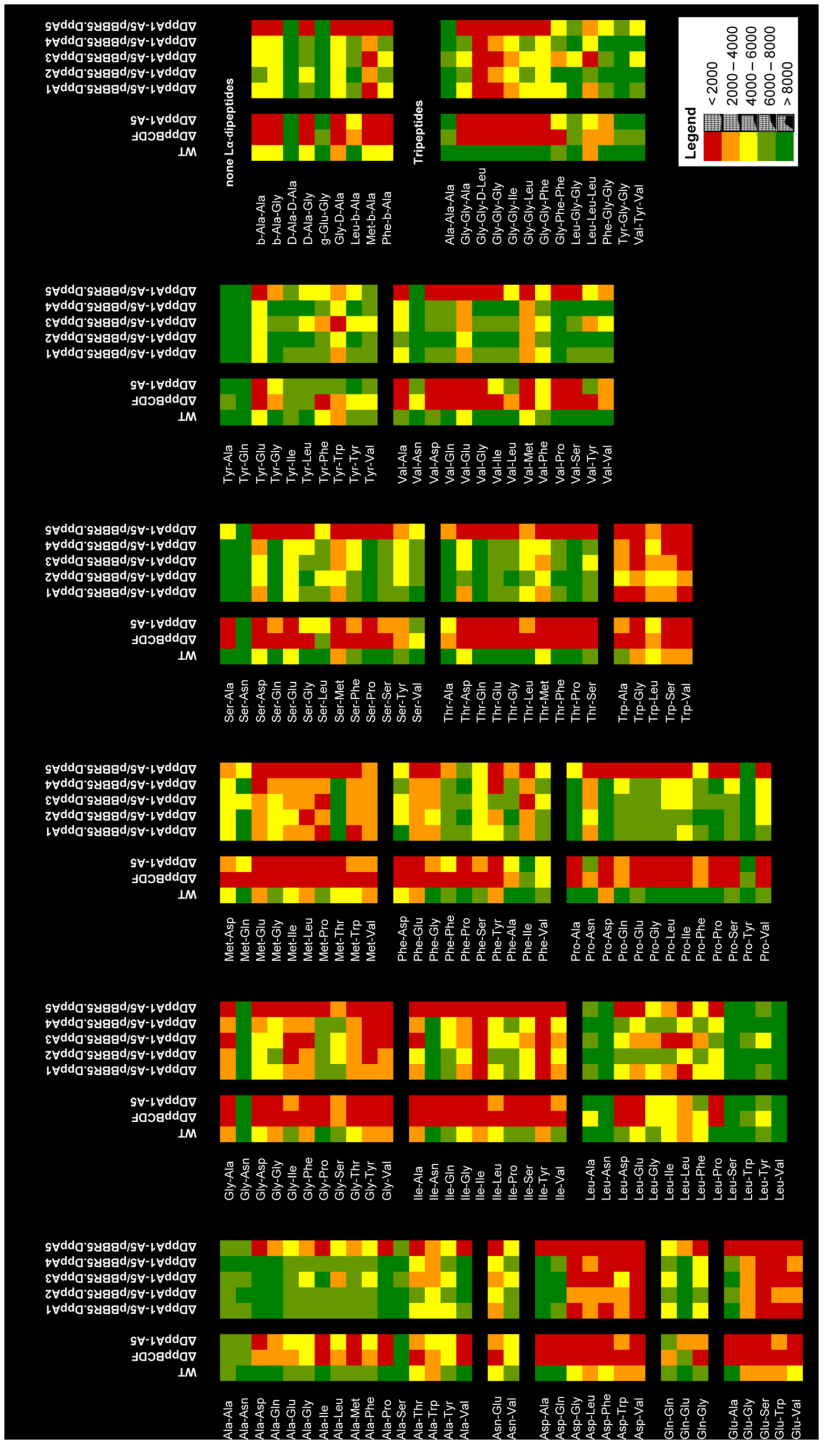
Heat map of di/tripeptide utilization by PA14, the DppBCDF transporter mutant, the SBP penta mutant DppA1–A5, and by strains of the penta mutant complemented with individual SBPs. Each square represents the average respiratory activity of a strain in one well of the Biolog Phenotype MicroArray plates. The heat map is based on the values reflecting the extent of respiration after 24 hours at 37°C. Values exceeding 8000 reflect solid respiratory activity during the assay. Values below 2000 were considered as no respiratory activity.

In order to investigate the substrate profile of the ABC transporter DppBCDF, a deletion mutant of the *dppBCDF* operon was constructed (Figure 1A). We used Biolog phenotype MicroArrays to elucidate the effect of the *dppBCDF* deletion on the ability of the mutant to utilize dipeptides. Dipeptides that did not support respiratory activity of the mutant indicated deficiency of the mutant to utilize the respective dipeptide as nitrogen source. Biolog respiration curve comparison between the wild type and the *dppBCDF*-deficient mutant for all PM plates are provided in Dataset S2. A direct comparison of utilized dipeptides in terms of the extent of respiratory activity is demonstrated in the heat map in Figure 4.

Numerous dipeptides could not be used as N-source by the mutant lacking the DppBCDF transporter system (as illustrated by the red colored squares in the heat map), identifying it as the main transporter system participating in the utilization of dipeptides. Overall, our data suggests that the transporter system is involved in the utilization of at least 66% of all dipeptides (107 out of 162) that are utilized by PA14 (Figure 3). The *dppBCDF*-deficient strain has a higher preference towards utilization of dipeptides containing acidic residues (29 out of 34), in contrast to dipeptides containing uncharged (40 out of 69) and nonpolar (95 out of 144) amino acids, respectively (Table 2). The utilization pattern of clustered dipeptides was not influenced by a N- or C-terminal localization of the amino acid residue. However, our data suggest that dipeptides coupled of uncharged-nonpolar (12 out of 22), nonpolar-uncharged (16 out of 29), and uncharged-uncharged (4 out of 9) residues are less efficiently taken up by the ABC transporter system (Table 1).

**Figure 5:**
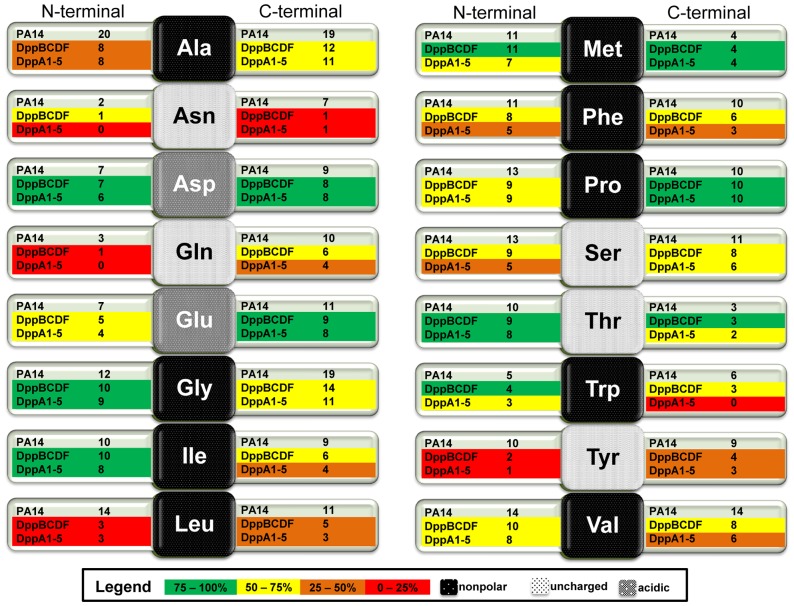
Effect of the localization of an amino acid residue on utilization of a dipeptide. The middle square boxes represent the specific amino acid. The background color of the box gives information about the chemical nature of the specific side-chain group. The left and right arms of a box indicate the localization of the amino acid either at the N- or C-terminal end of the dipeptide. The numbers within the arms indicate how many dipeptides of this group were utilized by the different strains. The colors within the arms indicate the percentage of dipeptides used by a strain (see legend) related to the total amount of dipeptides used by the wild type.

A closer look at single amino acid residues of the dipeptides revealed that only 1 out of 7 dipeptides that contained an Asn residue at the C-terminal end was utilized by the transporter (Figure 5). Moreover, only 2 out of 10 dipeptides containing Tyr at the N-terminal end could be used. Dipeptides containing Leu residues were poor substrates of the DppBCDF transporter. However, a localization of the Leu residue at the C-terminal end leads to a slightly increased utilization of these dipeptides by the ABC transporter. Moreover, less than 50% of dipeptides containing Ala or Gln at the N-terminal end were utilized by the transporter system (Figure 4, Figure 5). In contrast, almost all Thr-containing dipeptides were utilized regardless of their localization at the N- or C-terminal end. A similar observation was made with dipeptides containing Asp and Glu, which have negatively charged side chains. Several other dipeptides containing nonpolar amino acid residues such as Gly, Ile, Met, Pro, Thr, and Trp were good transporter substrates. While most of those residues were attached at the N-terminal end, Met and Pro showed higher preference of being utilized when residues were at the C-terminal end (Figure 5).

### Role of substrate-binding proteins in dipeptides utilization

In order to investigate the role of the SBPs DppA1–A5 in dipeptide utilization, a penta deletion mutant, lacking all five SBPs, was constructed and investigated using Biolog phenotype MicroArrays. Biolog respiration curve comparison between the wild type and the penta mutant for all PM plates are provided in Dataset S2. A list of the excluded dipeptides is provided in the heat map in Figure S5. A comparison of the dipeptide utilization profiles of the wild type, the *dppBCDF* transporter mutant and the SBP penta mutant is shown as heat map in Figure 4. The utilization patterns of the transporter mutant and the SBP mutant are very similar. Both mutants are unable to utilize a wide range of dipeptides.

Our data suggest that about 52% of the dipeptides (84 out of 162) are recognized and delivered by the SBPs to the according permease (Table 2). The observed phenotypes indicate a higher preference towards dipeptides containing acidic residues (26 out of 34), in contrast to dipeptides containing uncharged (28 out of 69) and nonpolar (75 out of 144) amino acids (Table 2).

The substrate profiles of the SBP penta mutant and the *dppBCDF* transporter mutant overlap with only a few exceptions. The SBP penta mutant is able to utilize more substrates than the transporter mutant indicating that additional proteins might be involved in the delivery of substrates to the permease. Approximately 15% less dipeptides were recognized by the SBPs when compared to total substrate spectrum of the ABC transporter (Table 1).

### Utilization of tripeptides as nitrogen source

The Biolog phenotype MicroArray plate PM8 contains 14 tripeptides as sole nitrogen sources. PA14 was able to use 13 tripeptides as nitrogen source (Figure 4). The tripeptide that PA14 could not use contains a D-form of alanine at the N-terminus (Figure S5).

Most of the tested tripeptides contain two glycine residues located either at the N-terminal or the C-terminal end. Tripeptides having two Gly residues at the N-terminal end are transported via DppBCDF and at least one is recognized by the SBPs. However, when the Gly-Gly is located at the C-terminal end, both the transporter mutant as well as SBP penta mutant showed still respiratory activity in the presence of the tripeptide, indicating that it is transported into the cells via a different transport mechanism or following proteolytic cleavage (Figure 4).

### Substrate specificity of the dipeptide-binding proteins

In order to elucidate the substrate specificity of the five SBPs associated with dipeptide transporter, we complemented the SBP penta mutant with plasmids carrying individual SBPs. Comparisons of the Biolog respiration curves of the SBP penta mutant and of the penta mutant complemented with an individual SBP are provided in Dataset S2. A comparison of respiration promotion by the dipeptides is shown in the heat map in Figure 4. Excluded dipeptides are shown in Figure S5. We used qRT-PCR to determine the expression levels of the SBPs in the SBP penta mutant carrying complementation plasmids. As expected, expression of the SBPs was at higher levels in the complemented strains when compared to their native expression in the PA14 wild-type strain during early exponential phase: DppA1 (13-fold), DppA2 (11-fold), DppA3 (35-fold), DppA4 (24-fold), and DppA5 (18-fold).

The heat map of Figure 4 shows that dipeptides containing negatively charged (Asp or Glu) or nonpolar amino acids (Gly, Ile, Met, or Trp) did not support respiratory activity of the penta mutant complemented with the different SBPs or they enabled only a low level of respiration.

**Figure 6:**
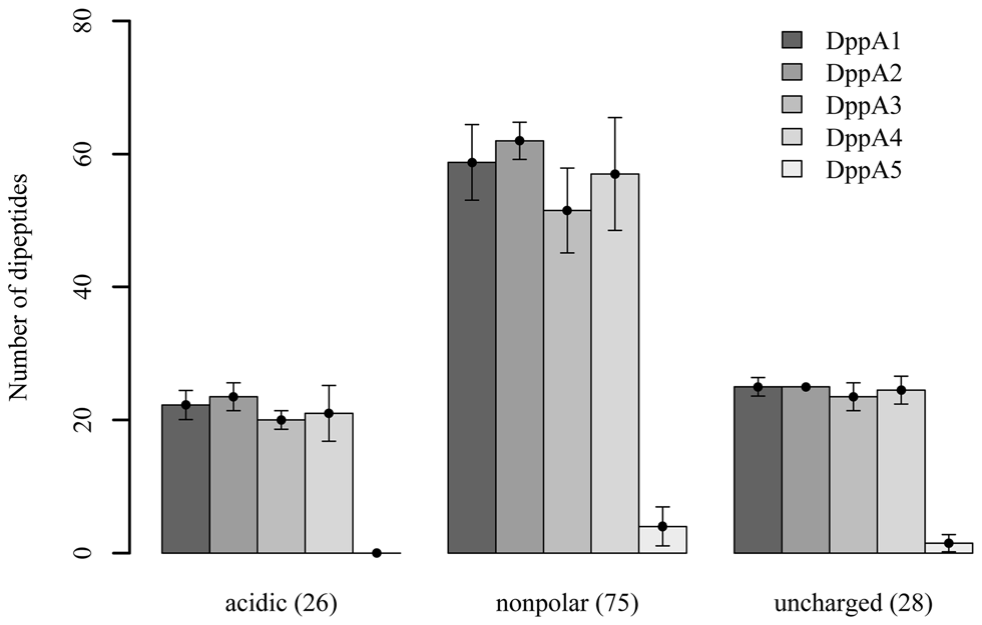
Utilization of dipeptides by strains of the SBP penta mutant complemented with individual SBPs. The numbers in parentheses indicate the total number of dipeptides analyzed. There are no significant differences among the complemented SBPs DppA1 to DppA4 as determined by single factor ANOVA. DppA1 to DppA4 show significant difference to DppA5 (*p*<0.05) as determined by a two-sided t test with equal variances.

We observed that all SBPs are able to bind and deliver dipeptides with different side chains to the ABC transporter. DppA2 was able to recognize more dipeptides than any other SBP, about 15% of these dipeptides contain acidic residues (Figure 6). We observed the tendency that all SBPs, except DppA5, have a higher affinity for dipeptides containing acidic residues at the C-terminus than for dipeptides containing acidic residues at the N-terminus.

**Figure 7:**
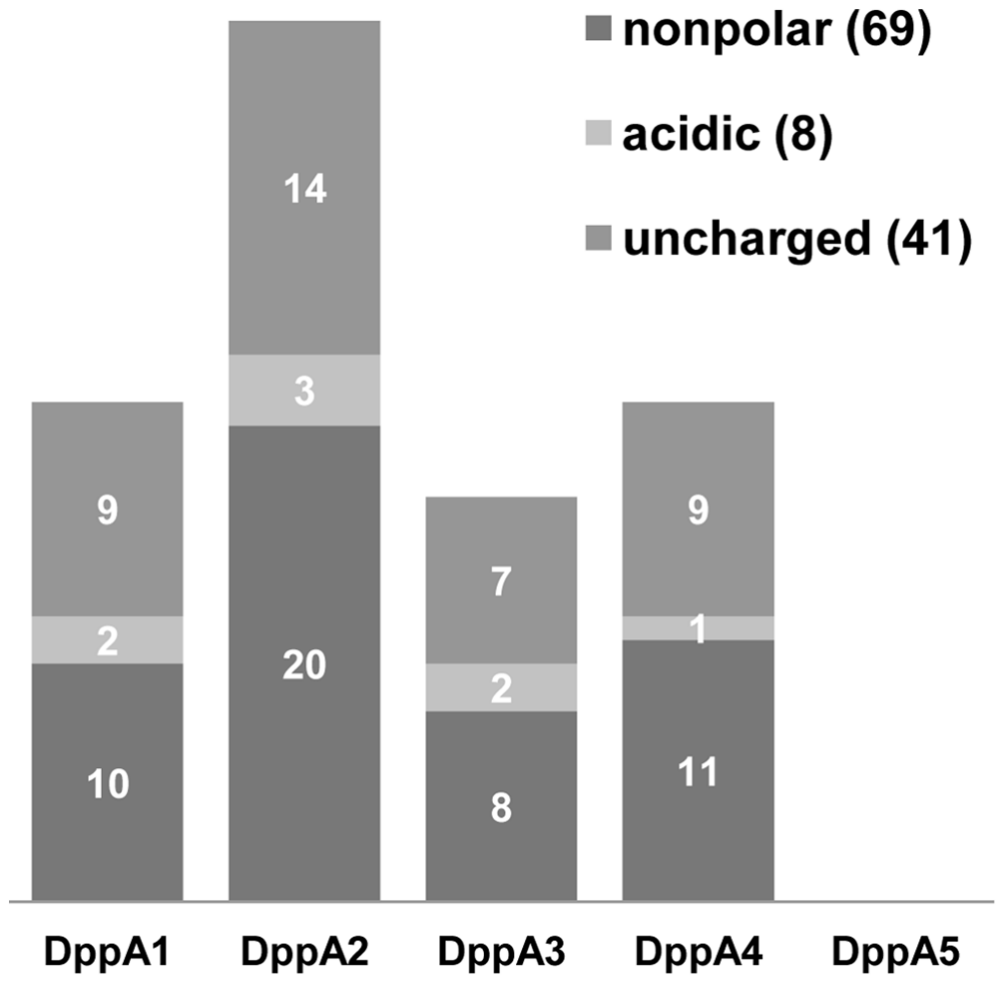
Effect of the chemical nature of amino acid residues located either at the N- or C-terminal end of dipeptides on the utilization by strains of the SBP penta mutant complemented with individual SBPs. The numbers indicate dipeptides that enabled a more than 2-fold increase in respiratory activity of SBP mutant complemented with an individual SBPs compared to the respiratory activity of the mutant carrying an empty plasmid. The SBP penta mutant was able to utilize these dipeptides.

The utilization of dipeptides was not strongly influenced by the type of amino acid residues located at the N- or C-terminal end. Although, it appears that DppA1, DppA3, and DppA4 prefer dipeptides with acidic residues at the C-terminal end (Table 3). Moreover, the SBP penta mutant was still able to utilize 118 clustered dipeptides as N source. In order to investigate whether the SBPs are able to increase the utilization of those peptides, we searched for dipeptides that increased the respiratory activity of the complemented strains at least 2-fold compared to the penta mutant. We found that DppA2 is able to increase the utilization of about 30% of all dipeptides, while the other SBPs were able to increase the utilization of less than 20% of dipeptides (Figure 7).

**Table 3:** Effect of side chain properties of the terminal amino acid on utilization of dipeptides by the SBP penta mutant complemented with plasmids harboring an individual dipeptide-binding protein.

Dipeptide utilization by SBPs^a^		2^nd^ amino acid															Total (N-terminal)				
		nonpolar					acidic					uncharged									
		A1	A2	A3	A4	A5	A1	A2	A3	A4	A5	A1	A2	A3	A4	A5	A1	A2	A3	A4	A5
1^st^ amino acid	nonpolar	32	34	27	32	1	11	11	11	10	0	10	10	9	9	0	53	55	47	51	1
	acidic	4	6	4	3	0	0	0	0	0	0	1	2	1	1	0	5	8	5	4	0
	uncharged	7	7	7	7	1	5	5	5	5	0	2	2	2	2	0	14	14	14	14	1
	Total (C-terminal)	43	47	38	42	2	16	16	16	15	0	13	14	12	12	0	72	77	66	69	2

a The number includes only dipeptides that were not used by the SBP penta mutant.

Furthermore, we determined the utilization efficiency of the different SBPs. Therefore, we compared the respiration of the penta mutant strains complemented with the different SBPs, considering differences more than 2-fold as significant. In total, we found almost 70 dipeptides showing a difference of at least 2-fold (Table 4). The strain expressing DppA2 was able to use 35 dipeptides more efficiently than the strains expressing other SBPs.

**Table 4:** Differences in dipeptide utilization efficiency of the SBPs DppA1–DppA4 determined by differences in total growth^a^.

		Increased utilization^b^			
		DppA1	DppA2	DppA3	DppA4
Decreased utilization^b^	DppA1	-	11	0	3
	DppA2	1	-	3	1
	DppA3	6	17	-	11
	DppA4	5	7	3	-

a DppA5 was omitted since it showed a similar phenotype as the SBP penta mutant.

b Number of dipeptides where strains complemented with different SBPs showed at least 2-fold differences in respiration.

Moreover, almost all tripeptides were more efficiently used by strains expressing DppA2 and DppA4 than by strains expression DppA1, DppA3, and DppA5 (Figure 4).

### Growth experiment with di/tripeptides as sole nitrogen source

Independent growth studies of batch cultures in minimal medium supplemented with di/tripeptides as sole nitrogen source were undertaken to confirm some of the respiration phenotypes of the strains from the Biolog phenotype MicroArray plates (Table 5). These growth assays confirmed most of the phenotypes of the PA14 strains, with some minor discrepancies with regard to the ability of specific substrates to act as sole nitrogen source. For example, the SBP penta mutant complemented with DppA3 showed a medium respiratory activity in wells of the Biolog plates containing the dipeptides Ala-Phe, Phe-Val, and Pro-Leu. However, the strain showed solid growth in minimal medium supplemented with these dipeptides.

**Table 5:** Growth of PA14, the SBP penta mutant, and strains of the penta mutant complemented with individual SBPs in minimal medium supplemented with di/tripeptides as the sole nitrogen source^a^.

N source	PA14 wt		ΔdppA1–A5		DppA1		DppA2		DppA3		DppA4	
	B^b^	OD_600_ c	B	OD_600_	B	OD_600_	B	OD_600_	B	OD_600_	B	OD_600_
Ala-Ala	++	0.55±0.02	++	**0.13±0.05** d	++	0.59±0.04	++	0.38±0.21	++	0.70±0.04	+++	0.43±0.24
Ala-Gln	+++	0.81±0.04	+/−	0.20±0.03	+++	0.81±0.03	+++	0.56±0.29	+++	0.83±0.04	+++	0.55±0.30
Ala-Phe	++	0.49±0.01	+	0.10±0.01	++	0.50±0.06	++	0.24±0.14	+	**0.67±0.01**	++	0.27±0.16
Phe-Pro	+++	0.65±0.04	−	0.03±0.03	++	0.56±0.03	+++	**0.13±0.08**	++	0.60±0.04	+++	0.21±0.13
Phe-Val	++	0.39±0.10	+	0.19±0.05	++	0.39±0.05	++	0.27±0.06	+	**0.58±0.13**	++	0.31±0.09
Pro-Gly	+++	0.45±0.03	−	0.09±0.01	++	0.53±0.04	++	0.29±0.18	++	0.68±0.07	++	0.26±0.21
Pro-Leu	+++	0.30±0.02	−	0.06±0.03	++	0.21±0.03	++	**0.11±0.05**	+	**0.70±0.03**	+	0.10±0.01
Thr-Gln	+++	0.57±0.20	−	0.09±0.07	+++	0.56±0.02	+++	0.25±0.03	+++	0.78±0.01	+++	0.37±0.04
Gly-Gly-Gly	+++	**0.14±0.05**	−	0.03±0.02	−	0.08±0.02	+/−	0.11±0.04	−	0.07±0.05	+/−	0.11±0.04
NH_4_Cl		0.63±0.08		0.50±0.14		0.57±0.03		0.54±0.08		0.51±0.12		0.60±0.01

a DppA5 was omitted since it showed a similar phenotype as the SBP penta mutant in the Biolog Phenotype MicroArrays.

b Average respiratory activity of a strain in one well of the Biolog Phenotype MicroArray plates after 24 hours. −, <2000 (no respiratory activity); +/−, 2000–4000; +, 4000–6000; ++, 6000–8000; +++, >8000 (solid respiratory activity).

c Optical densities were determined after 24 h of growth in Minimal Medium P supplemented with 2 mM of a di/tripeptide or NH_4_Cl as sole nitrogen source.

d Boldface numbers indicate a significant difference between the extent of respiratory activity in Biolog Phenotype MicroArray and the extent of growth in minimal medium supplemented with di/tripeptides as the sole nitrogen source.

### Uptake of phaseolotoxin by the DppBCDF ABC transporter

The tripeptide phaseolotoxin [(N δ-phosphosulfamyl)ornithylalanylhomoarginine] is produced by the plant pathogen *P. syringae* pv. *phaseolicola*, and shows phytotoxic and antibacterial activity. It has been demonstrated that oligopeptide transporters are responsible for the uptake of this toxin into bacterial cells [37]. This prompted us to investigate whether *P. aeruginosa* PA14 is susceptible towards phaseolotoxin and whether the dipeptide transporter is involved in the uptake of phaseolotoxin.

Our results demonstrate that PA14 is sensitive to phaseolotoxin and that it is specifically taken up by the dipeptide ABC transporter DppBCDF. This finding is interesting since other bacteria use an oligopeptide transporter [37] and not a dipeptide transporter for the uptake of phaseolotoxin.

**Figure 8:**
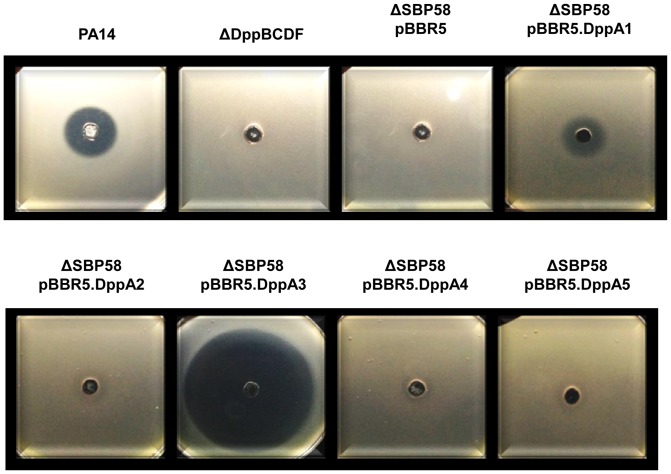
Growth inhibition of PA14, the dipeptide transporter mutant, the SBP penta mutant and strains of the SBP penta mutant complemented with individual SBPs by phaseolotoxin.

Furthermore, we elucidated which SBP is responsible for the delivery of phaseolotoxin to the ABC transporter. Our results demonstrate that DppA3 is involved in the transport of the toxin to the Dpp permease, as can be seen by the increased growth inhibition of the strain expression DppA3 caused by phaseolotoxin (Figure 8). In addition, DppA1 is also involved in the uptake of phaseolotoxin.

## Discussion

Microorganisms need to take up nutrients from the environment as an inevitable consequence for survival and growth. Proteins, if available in adequate supply, are a major source of carbon and nitrogen molecules. However, due to the size limitation of molecules that can permeate the bacterial cell envelope, large proteins have to be degraded by extracellular proteases [38] before they can be utilized by the organism.

*P. aeruginosa* produces and secretes several peptidases, such as elastase A (LasA) that cleaves the pentaglycine bonds in the peptidoglycan of *Staphylococcus aureus* [39], elastase B (LasB) that cleaves peptide bonds on the N-terminal end of hydrophobic residues [40], alkaline proteinase AprA [41], the lysine-specific endopeptidase protease IV [42], the endoprotease PrpL [43], the leucine-specific aminopeptidase PaAP [44], [45], and an arginine-specific aminopeptidase [25].

Once proteins are cleaved into small peptides and/or amino acids, they can be readily transported into the cell. In pseudomonads, uptake through the outer membrane is almost exclusively via specific porins [24]. Once in the periplasm, specific transporter systems are required to translocate the molecules into the cytoplasm, where they are utilized as energy source [44].

The key objective of this study was to elucidate and characterize the specific uptake system of dipeptides in *P. aeruginosa*. We determined the substrate specificity of the dipeptide ABC transporter system DppBCDF and its associated substrate-binding proteins DppA1–A5. Therefore, we utilized the phenotypic MicroArray-based high-throughput screening method from Biolog that uses cellular respiration as a reporter. Using this approach, we were able to demonstrate that the DppBCDF transporter system is clearly responsible for the uptake and utilization of various di/tripeptides and we provide for the first time an extended overview of di/tripeptide substrates for this transport machinery. Additionally, we delineate a large pattern of various di/tripeptides that are recognized by the associated substrate-binding proteins. Such a high-throughput screening method to characterize the substrate specificity of the DppBCDF transporter system from *P. aeruginosa* has hitherto not been carried out.

### The transport machinery

In order to study the substrate specificity of the dipeptide transporter system, we determined the dipeptide utilization in a mutant lacking the uptake machinery. However, our data revealed that several dipeptides could also be utilized by a *dppBCDF*-deficient mutant. When we examined these dipeptides, we found that specific aminopeptidases may contribute to peptide utilization by cleaving dipeptides into single amino acid residues. Hence, our screening approach had some restrictions. One example are Ala-containing dipeptides, where it has previously been reported that aminopeptidase N is involved in the degradation of Ala-Ala dipeptides [46]. We found in our screening assay, that most Ala-containing dipeptides could be utilized by the *dppBCDF*-deficient strain; however, with lower efficiency compared to the wild type (Figure 4). This may correlate with the aforementioned involvement of peptidase N. Similar phenotypes were observed with dipeptides containing Leu, Tyr, Gln (Figure 4) as well as Arg, Lys, and His (Figure S5).

Nevertheless, the deletion of the dipeptide transporter abolished the ability of PA14 to use a broad range of dipeptides, which could otherwise serve as energy source (Figure 4). It has been reported that the Dpp transporter system is able to recognize and transport various dipeptides unrelated to their side chains [16]. Our screening approach provides an overview of almost 200 dipeptides with different side chains that are translocated via the dipeptide permease. Our data suggest that the transporter system prefers acidic side chains over nonpolar or uncharged ones (Table 2). In general, it appears that there is no clear preference for side chains located at the N- or C-terminal end. Although some amino acid residues might be preferred at a specific position, such as Gly, Ile, Thr, and Trp at the N-terminal end and the hydrophobic Met and Pro at the C-terminal end (Figure 5). Our screening approach also showed that dipeptides composed of uncharged and nonpolar residues are poor substrates of the DppBCDF transporter (Table 1).

With the few dipeptides that contain a D-form amino acid and are present in the Biolog plates, it became apparent that the sterochemical preference of the dipeptide transporter is for L-form residues. Almost none of the D-form dipeptides could be utilized by PA14 (Figure S5), a result in accord with previous studies in *E. coli* [16], [21], [47].

Several studies have shown that dipeptide ABC transporters from other bacteria are able to transport also other compounds beside dipeptides. It has for example been demonstrated that a dipeptide transporter mutant of *Helicobacter pylori* was not able to utilize dipeptides as well as hexa- and nonapeptides [17]. The heme precursor 5-aminolaevulinic acid is a specific substrate of the dipeptide transporter from *E. coli* and *S. typhimurium* [23], [48]. Moreover, ions such as nickel have been associated with dipeptide transporter systems [49]. The eukaryotic di/tripeptide transporter Dal5p from *Saccharomyces cerevisiae* is able to transport the nitrogenous compound allantoate/ureidosuccinate [50]. Interestingly, allantoin, the none-hydrolyzed form of allantoate, appears to be transported by the DppBCDF system in *P. aeruginosa* as observed in our screening (Figure S5).

### The periplasmic delivery system

Substrate-binding proteins accomplish the recognition and binding of substrates in the periplasm and determine the substrate specificity of the associated ABC importer. The amount of SBPs varies among organisms. For example the dipeptide ABC transporter of *E. coli* possesses only one SBP, while *Pseudomonas putida* has two, and *Pseudomonas fluorescence* three SBPs [36].

The question arises why does the *P. aeruginosa* ABC dipeptide transporter possess even five SBPs? Do multiple SBPs have different binding specificity, and therefore broaden the specificity of the ABC transporter? If these SBPs occurred through gene duplication, they might have acquired a novel cellular function, thus providing additional capability to utilize nutrients. Since duplications will also share some common features, multiple SBPs would be able to complement each other in case of mutational inactivation or gene loss. Hence, we hypothesized that the five present SBPs may be biologically relevant in terms of the recognition of specific di/tripeptides, and therefore increase peptide transport efficiency and optimize nutrient utilization.

To study the impact of each SBP, we constructed a penta mutant of PA14 lacking all five SBPs, complemented the mutant with individual SBP and used these strains in our Biolog screening assay. We retrieved extensive information about the substrate specificity. However, our data suggests that several SBPs have no clear preference for dipeptides with a specific side chain. It became apparent that some dipeptides can be utilized with different affinity by various SBPs. A possible reason may be the fact that the binding proteins recognize rather the peptide backbone than the connected side chains [51]. This is in contrast to specific amino acid receptors, which specifically recognize the side chain [52]. With respect to tripeptides tested in our assay, we identified DppA2 and DppA4 to be more efficient in tripeptide usage than the other SBPs (Figure 4).

SBPs tolerate binding of structurally different dipeptides by recognizing the backbone of the dipeptide while leaving enough space for a variety of side chains [53]. The structure of SBPs consists of two domains that are connected by two or three strands, which act as hinge, forming a pocket-like structure. The actual binding site is located in the groove between the two domains and appears to be large enough to bind any side-chain [52], [53]. However, SBP specificity can change by variation of the structure of the active site in the side chain-binding pockets [54]. Specific acidic and basic residues inside the pocket interact with the N- and C-terminal end of peptides by formation of salt bridges [55]. Once the peptide is bound, the SBP will close itself by bending the hinge, thus engulfing the peptide. An interaction with the substrate-binding domain of the permease, and subsequent initiation of nutrient translocation, is only possible in a closed form [52], [53].

The transfer of the substrate to the permease might be another restriction that influences the specificity. It has previously been demonstrated that ABC transporters have one or more substrate-binding domains in close proximity to the translocation pore, which interact with SBPs. In our assay, the penta mutant lacking all five SBPs, was still able to utilize more dipeptides than the DppBCDF transporter mutant indicating that additional proteins are involved in the transport of the dipeptides or that some of the dipeptides can be directly transported by the ABC transporter. This has been demonstrated for maltose when the transporter machinery was mutated [56].

It has previously been reported that the presence of multiple, independently regulated SBPs increases the substrate spectrum of the associated transporter and might be involved in the adaptation of the bacterium to changing environmental conditions [20], [57]–[59]. The uptake of specific peptides can trigger bacterial adaptations such as chemotaxis and quorum sensing [16]. In this connection it has been shown that DppA1 is a quorum sensing-related gene in *P. aeruginosa* [60]. DppA3 has been demonstrated to be iron-regulated with increased expression under iron-limiting conditions [61]. Moreover, mutants defective in DppA1, DppA2, and DppA4 were found to exhibit poor biofilm formation when compared to the wild-type strain [48].

Using the available RNA-seq data for *P. aeruginosa*, we found that DppA2 and DppA5 are only expressed at a low level [35]. Whether the expression of DppA2 and DppA5 is induced under specific conditions, needs to be addressed in further experiments. Studies in *Borrelia burgdorferi* demonstrated that the SBP OppA-5 is temperature-regulated [62] and that OppA-4 and OppA-5 were highly up-regulated in a mouse model [55]. Further experimental studies in a host-related environment may identify the specific induction conditions of the dipeptide-binding proteins of *P. aeruginosa* PA14. The transcription of DppA4 in an operon with the outer membrane porin OpdP might hind towards a more specific role of this SBP. The porin OpdP was previously shown to be involved in the transport of dipeptides as well as of single amino acids in PAO1 [24].

Overall, our data provide insights into the substrate specificity of the five SBPs from *P. aeruginosa*. Individually regulated SBPs, activated under certain conditions, may give an advantage in adapting to various environmental conditions. We have shown that four of the five SBPs have overlapping substrate profiles, where DppA2 shows the highest flexibility, tolerating the binding of more peptides than the other SBPs. This is interesting since it appears that DppA2 is only expressed at a low level under *in vitro* growth conditions. Moreover, we found that the various SBPs of the dipeptide transporter can complement each other, while it has been shown that the periplasmic binding proteins OppA and OppB of the oligopeptide transporter from *P. aeruginosa* cannot compensate for each other. Both SBPs were required for uptake of uridyl peptide antibiotics, indicating that OppA and OppB may form an oligomeric binding protein [32].

### Fraud translocation

Peptide transporters have the ability to recognize and transport a broad range of substrates. Hence, they became attractive targets for therapeutic agents [63]. In nature, various microorganisms produce antimicrobial substances in order to inhibit the growth of competing organisms. Often such compounds are peptide analogues and have been demonstrated to penetrate the cell via peptide transporters [9], [37]. It has recently been shown that the oligopeptide transporter OppCDE of *P. aeruginosa* PAO1 is required for uptake of uridyl peptide antibiotics and for import of the tripeptide herbicide bialaphos [32].

Phaseolotoxin, a highly charged, phytotoxic tripeptide produced by *Pseudomonas syringae* pv. *phaseolicola*, is able to inhibit the enzyme ornithine carbamoyltransferase (OCTase) of various organisms [37], [64]. Previous studies have demonstrated the toxic effect in *E. coli* and *S. typhimurium* strains, where it was shown that the toxin gets access to the cell through the uptake via an oligopeptide permease [37]. The closest homolog of the oligopeptide transporter from *E. coli* in PA14 is the dipeptide transporter DppBCDF.

To our knowledge, there are no data available about the toxic effect of phaseolotoxin on *P. aeruginosa*. It has been suggested that it is inactive against many *Pseudomonas* spp. [37]. However, we found that the toxin is able to inhibit growth of *P. aeruginosa* PA14 and we were able to demonstrate a lack of toxicity in the DppBCDF transporter mutant as well as in the SBP penta mutant (Figure 8). Moreover, we identified that the SBPs DppA1, and with much greater extend DppA3, are able to deliver the toxin to the permease. Interestingly, these two proteins show also the highest sequence similarities to the *E. coli* OppA protein.

## Materials and Methods

### Bacterial strains, plasmids and growth conditions

All used nucleotide sequences were based on the genome of *P. aeruginosa* UCBPP-PA14 (GenBank: NC_008463.1) available from NCBI, hence referred to as PA14 in this manuscript. Bacterial strains are listed in Table 6 and plasmids in Table S1.

**Table 6:** Bacterial strains used in this study.

Strain	Relevant characteristics or genotype^a^	Reference or source
***Escherichia coli***		
XL1-Blue	*recA*1 *endA*1 *gyrA*96 *thi-1 hsdR*17(r_K_^−^ m_K_^+^) *supE*44 *relA*1 *lac* [F' *proAB lacI* ^q^ ZΔM15Tn*10*(Tc^r^)]	Stratagene
ST18	*pro thi hsdR^+^* Tp^r^ Sm^r^; chromosome::RP4-2 Tc::Mu-Kan::Tn7/λpir Δ*hemA*	[83]
***Pseudomonas syringae***		
pv. phaseolicola 6/0	Wild type from bush bean, producer of phaseolotoxin	[73]
***Pseudomonas aeruginosa***		
PA14	Wild type	[84]
PA14.ΔdppBCDF	*dppBCDF* deletion mutant	This study
PA14.ΔdppA1-A5	*dppA1 dppA2 dppA3 dppA4 dppA5* penta deletion mutant	This study
PA14.ΔdppA1-A5 (pBBR5. DppA1)	SBP penta mutant complemented with *dppA1*	This study
PA14.ΔdppA1-A5 (pBBR5. DppA2)	SBP penta mutant complemented with *dppA2*	This study
PA14.ΔdppA1-A5 (pBBR5. DppA3)	SBP penta mutant complemented with *dppA3*	This study
PA14.ΔdppA1-A5 (pBBR5.S DppA4)	SBP penta mutant complemented with *dppA4*	This study
PA14.ΔdppA1-A5 (pBBR5.S DppA5)	SBP penta mutant complemented with *dppA5*	This study

a Antibiotic resistance: Kan, kanamycin; Sm^r^, streptomycin, Tc, tetracycline; Tp^r^, trimethoprim.

*P. aeruginosa* strains were cultured at 37°C in Luria Bertani (LB) broth and Minimal Medium P (MMP) [25]. Selection for transformed pseudomonads was achieved on King's B medium. *E. coli* XL-1 Blue was used as cloning host. *E. coli* ST18 was used for biparental mating where the medium was supplemented with 50 µg/ml 5-aminolevulinic acid (ALA). *E. coli* cells were routinely maintained at 37°C in dYT medium, except when strains were used that contained the FLP recombinase (30°C).

When required, cultures were supplemented with 50 µg/ml ampicillin (Ap), 25 µg/ml chloramphenicol (Cm), or 20 µg/ml gentamicin (Gm) for *E. coli* or 500 µg/ml carbenicillin (Cb) and 100 µg/ml gentamicin (Gm) for PA14. Bacterial growth was monitored using a spectrophotometer at 600 nm (OD_600_).

### PCR amplifications and DNA modifications

PCR primers are listed in Table S2. Screening PCR reactions were carried out using DreamTaq DNA polymerase (Thermo Scientific) in accordance with the manufacturer's instructions and optimized annealing temperature for each primer set. For screening PCR reactions performed with PA14, bacterial cells were boiled at 95°C for 5 min and subsequently pelleted at 13,000 rpm for 1 min. PCR reactions were supplemented with additional 5% DMSO. For high fidelity PCR reactions, Phusion DNA polymerase (Thermo Scientific) or Q5 DNA polymerase (NEB) were used.

Restriction digestions were performed using Thermo Scientific restriction enzymes according to the manufacturer's instructions at the appropriate temperature. All ligation reactions were carried out at room temperature using Thermo Scientific T4 DNA ligase.

DNA purifications were either performed using the GeneJET PCR purification kit (Thermo Scientific) or the GeneJET Gel extraction kit (Thermo Scientific) following the manufacturer's instructions.

### Construction of the PA14 *dppBCDF* knockout mutant

The construction of the knockout vector was based on the protocol described by Zumaquero *et al.* [65]. Briefly, approximately 500 bp flanking the 5′ and 3′ regions of the *dppBCDF* dipeptide ABC transporter operon were PCR-amplified using the primer pairs 58420-A1/58420-A2 and 58490-B1/58500-B2. The T7 primer sequence was incorporated in primers A2 and B1 to provide homology and a *Kpn*I restriction site between the fragments. After amplification, the obtained fragments were gel-purified and approximately 40 ng of each fragment was used in a polymerization PCR reaction with primers A1 and B2. The resulting fusion product was gel-purified, further ligated into the pGEM-T easy vector and verified by sequencing.

A gentamicin cassette, coupled to GFP and flanked by Flp-*FRT* sites, was cut from plasmid pPS858-Eco and subsequently inserted into the *Kpn*I digested pGEM construct. The deletion allele was cut and further ligated into *Eco*RI-digested pEX18Ap, yielding the final replacement plasmid pEX18Ap.ABC58-ko.

The generation of the PA14 *dppBCDF* mutant was based on the published procedure from Schweizer and Hoang [66]. Briefly, chromosomal deletions were obtained by conjugational transfer of the gene replacement vector into PA14. Therefore, bacteria, grown overnight on agar plates, were scratched from the plate, resuspended in 1 ml of sterile water, and adjusted to an OD_600_ of 0.1. 100 µl of the *E. coli* ST18 donor strain, harboring the replacement vector, was mixed with 200 µl of the *P. aeruginosa* strain. The complete mixture was spotted (50 µl per spot) on a KB agar plate containing ALA and allowed to grow overnight. On the next day, bacteria were scratched from each spot, pooled, resuspended in 1 ml sterile water, and serially diluted. 100 µl of each dilution was spread on KB plates containing the appropriate antibiotic. On the next day, single colonies were picked on KB plates containing 5% sucrose to counter-select mutations from single crossovers. Remaining cells were pooled and re-streaked on 5% sucrose plates. From the next day onwards, putative mutants were screened for homologous recombination events by testing their antibiotic resistance on KB plates containing Gm and Cb, respectively. In order to confirm gene deletion through a double crossover event in Gm-resistant and Cb-sensitive colonies, primers were designed, which bind up- and downstream of the operon. PCRs were done using these locus-specific primers with primers binding within the Gm-GFP cassette.

The Gm-GFP-*FRT* cassette was finally excised using the plasmid pFLP2 that carries the FLP recombinase gene [67], [68]. Briefly, plasmid pFLP2 was conjugationally transferred into Gm-resistant mutants and selected at 30°C on KB plates containing Cb. Single colonies were selected and tested on agar plates containing Gm to confirm successful excision of the Gm-GFP cassette. A PCR with the locus-specific primers was performed. Sequencing of the obtained PCR fragment confirmed loss of the *dppBCDF* operon. Subsequently, loss of plasmid pFLP2 was achieved by incubating the mutants on 5% sucrose-containing agar plates. Loss of pFLP2 plasmid was confirmed by incubating the mutants on plates containing Cb.

### Construction of the substrate-binding protein (SBP) penta mutant of PA14

In order to delete the five SBPs (DppA1–DppA5) of the dipeptide transporter DppBCDF, a step-wise deletion of each SBP was accomplished. Therefore, we used a gene replacement strategy to create unmarked mutants, which had previously been described [67]. Briefly, flanking regions of the SBP PA14_58350 (DppA1), PA14_58360 (DppA2), PA14_58390 (DppA3), PA14_58420 (DppA4) and PA14_70200 (DppA5) were PCR amplified (Table S2) and gel-purified. By following the same procedure as described above, the obtained fragments were fused in an overlapping PCR reaction. Next, the fusion fragments were cloned into the suicide vector pEX18Gm via *EcoR*I/*Hind*III restriction sites, verified by sequencing, mobilized into PA14 and screened for double crossover events.

### Construction of SBP overexpression plasmids

Computational promoter prediction was based on the available RNA-seq dataset for *P. aeruginosa* [34] and the prokaryote promoter prediction tool PePPER [34]. The full ORFs including their promoter region were PCR amplified, cloned into pBBR1MCS-5 [69] and subsequently sequenced to rule out any possible mutations that might have occurred during PCR. To obtain expression from the native promoter, all constructs were cloned in opposite direction of P_lac_.

Briefly, a 1974-bp fragment containing *dppA1*, including the upstream promoter region, was amplified using primer SBP+Pro_58350_fwd(SacI)/SBP+Pro_58350_fwd(ApaI) and cloned into *Sac*I/*Apa*I-digested pBBR1MCS-5, yielding pBBR5.SBP58350-Pro. Construction of pBBR5.SBP58360-Pro, pBBR5.SBP58390-Pro and pBBR5.SBP70200-Pro was performed similarly. Since *dppA4* is found in an operon with the outer membrane porin PA14_58410, we fused the promoter region of the operon to the coding sequence of *dppA4* by an overlap PCR [70]. Briefly, the 241-bp promoter region was amplified using primers SBP+Pro_58420_fwd(XbaI)/SBP+Pro_58420_rev and the 1605-bp *dppA4* gene was amplified using primers SBP+Pro_58420_fwd/SBP+Pro_58420_rev(ApaI). The reverse primer of the promoter region and the forward primer of the gene contain a 22-bp overlap. Fragments were PCR-amplified and purified. Next, 6 ng of the promoter region and 40 ng of the *dppA4* gene were used to perform an overlapping PCR. The amplified product was gel-purified and an 1846-bp fragment was cut from the gel, digested with *Xba*I and *Apa*I, and subsequently cloned in *Xba*I/*Apa*I-digested pBBR1MCS-5, yielding plasmid pBBR5.SBP58420-Pro. All obtained constructs were verified by sequencing before they were mobilized into PA14 via biparental conjugation.

### RNA isolation and quantitative real-time PCR

Bacterial cultures were grown in KB broth until an optical density of 0.5. An aliquot containing 15×10^9^ CFU (equivalent of 15 ml OD_600_ of 1.0) was transferred to 15 ml killing buffer [71] and centrifuged for 20 min at 4000 rpm. The supernatant was decanted and the pellet frozen at −80°C.

Total RNA was isolated using the GeneJET RNA Purification Kit (Thermo Scientific) following the manufacturer's instructions. The obtained RNA was DNAse-treated (Ambion/Life Technologies) and subsequently checked for purity by gel electrophoresis and determination of the A_260_/A_280_ and A_260_/A_230_ ratios using a Nanodrop ND-2000 spectrophotometer (Thermo Fischer Scientific). High quality RNA was reverse transcribed and amplified with the OneStep RT-PCR Kit according to the manufacturer's protocol (Qiagen). Template RNA (5 ng) was used in a standard 25-µl qRT-PCR reaction with specific primers (Table S2). As control, RNA samples without reverse transcriptase were included to detect possible DNA contaminations.

For analysis, a Mastercycler ep *realplex* ^2^ gradient S (Eppendorf, Hamburg, Germany) was used. Cycling parameters included a 15 min initial denaturation at 95°C to activate the DNA polymerase followed by 40 cycles consisting of 15 sec at 95°C, 30 sec at 55°C and 30 sec at 72°C. The final step consisted of 1 min at 95°C and 30 sec at 55°C. A melting curve analysis with a temperature ramp from 25°C to 95°C in 20 min was performed at the end of each run to determine specificity of amplified qPCR products. Each sample was analyzed for gene expression in triplicate. Quantification of mRNA transcripts was performed by the comparative C_t_ method. Briefly, the C_t_ values of the samples of interest were compared with a non-treated sample. All C_t_ values were normalized to the *rpsL* gene [72]. The comparative C_t_ method was calculated by 2^−(ΔCt, sample−ΔCt, reference)^, where ΔC_t_ was normalized to the *rpsL* gene. Subsequently, fold-changes between the samples were determined based on the calculated C_t_ method.

### Phaseolotoxin agar diffusion assay

Phaseolotoxin was obtained from supernatants of stationary phase grown *P. syringae* pv. *phaseolicola* 6/0 cells [73]. Cells were grown in 5b medium (2.6 g KH_2_PO_4_, 5.5 g Na_2_HPO_4_, 2.5 g NH_4_Cl, and 1 g Na_2_SO_4_, 10 g glucose, 0.1 g MgCl_2_·6H_2_O and 0.01 g MnSO_4_·4H_2_O per liter [74]) at 18°C for 48 h and harvested by centrifugation at 4000 rpm for 20 min. The supernatant containing phaseolotoxin was used in an agar diffusion assay to evaluate the toxic activity of phaseolotoxin against PA14 and its mutant strains.

For preparation of the test plates, *P. aeruginosa* strains, grown on 5b medium (or MMP) at 37°C overnight, were resuspended and adjusted to an OD_600_ of 1.0 in sterile water. 500 µl of the cell suspension was added to 50 ml of 5b agar medium warmed to 50°C (or MMP). After preparing the plates, 7 mm holes were punched into the agar and 50 µl of the phaseolotoxin supernatant was added into the holes. Plates were incubated for 24 h at 37°C and subsequently visually analyzed in terms of growth inhibition zones on the bacterial lawn.

### Phenotype MicroArrays (Biolog)

The Biolog system for Phenotype MicroArrays (PMs) allows the measurement of bacterial phenotypes by monitoring cellular respiration using the irreversible reduction of tetrazolium dye to the purple compound formazan [75]. We utilized plates PM6, PM7 and PM8 to monitor catabolism of the implemented peptides as nitrogen sources by PA14 and its mutant strains (see Dataset S1 for details).

Prior to inoculation into PM plates, bacteria were grown on BUG agar plates overnight at 37°C. Using a sterile swab, cells were transferred into a sterile capped tube containing 16 ml of 1x IF-0 inoculating fluid and adjusted to a transmittance of 42% using a Turbidimeter (Biolog). 15 ml of the prepared cell suspension was added to 75 ml of 1x IF-0 and dye mix A to obtain a final transmittance of 85%. Additionally, for inoculation of plates PM6 to PM8, 2 M sodium succinate/200 µM ferric citrate (100x) was added to the suspension to obtain a 1x concentration. 100 µl of the cell suspension was inoculated into each well of the PM microplates. All plates were placed in the OmniLog reader (Biolog) and incubated for 24 h at 37°C. The OmniLog reader takes pictures of each plate at a 15-min time interval and converts the pixel intensity of each well into a signal value.

As a control, we incubated plates PM6, PM7 and PM8 with IF-0 media and dye mix A, but without bacteria. No significant dye reduction was observed without bacteria.

### Growth experiments with di/tripeptides as sole nitrogen source

We performed growth experiments with selected di/tripeptides as sole nitrogen source to validate that peptides that promoted bacterial cell respiration in the Phenotype MicroArrays promoted actually growth of the bacterium. Briefly, overnight cultures grown in Minimal Medium P (MMP) [25] were harvested by centrifugation and washed twice with phosphate-buffered saline (PBS) to remove any nitrogen source. Next, the cell suspension was adjusted to an OD_600_ of 0.05 with MMP supplemented with 2 mM of a di- or tripeptide as sole nitrogen source. Cultures were incubated at 37°C for 24 h in a shaking incubator at 230 rpm. Growth of the bacteria was determined using a spectrophotometer to measure the OD_600_. The experiment was repeated three times.

### Data analysis of the di/tripeptide utilization

Data were collected and analyzed with the OmniLog software and subsequently exported to Microsoft Excel [76]. All experiments were performed by a minimum of three replicates. Obtained data that were used in this study are provided in Dataset S1 (Excel).

Outliers among all replicates were identified by means of the standard deviation. Based on the difference of the standard deviation to the average value, we defined a threshold maximum of 20% deviation to be a good replicate. Samples with deviations between 20–50% were considered as acceptable. A deviation of more than 50% disqualified the sample from further analysis.

Respiratory activity of the cells in the single wells of the PM plates was quantified by a kinetic plot of color formation against time. The resulting area of each plot was represented by a specific data value. The threshold of this area value was set to 2000 (approximately 15–20% of the average area value of the positive control of all plates). In order to assist visualization of the data, we created a heat map based on data values representing the area under the respiration curve (subdivided into steps of 2000). Image comparisons of signal curves are provided in Dataset S2.

Next, the average values of all replicates were exported to Dataset S3 (Access) [76] in order to facilitate data retrieval and investigation of dipeptides in more detail. For every exported strain, one table was generated where the PM plate and the according well formed the primary key. Additionally, a table ‘chemical’ was generated, with the same primary keys as above, containing the chemical per well as well as every di/tripeptide split into single amino acid residues, their single core structure (alpha, beta, gamma) and form (L- or D-). Another table ‘AA’, with aa as primary key, was used to assign every amino acid to their specific side-chain residue.

Homann *et al.* [12] recommended to omit peptides containing lysine, histidine, and cysteine residues since they appear to support reduction of the dye in the presence of cells, but in the absence of growth [12]{Homann, 2005 #25}. Furthermore, the PM6, PM7 and PM8 plates contain only two cysteine-containing dipeptides (Cys-Gly and Gly-Cys), which supported only very low or no dye reduction by the PA14 strains. Hence, we excluded those peptides from our data analysis. Furthermore, due to the arginine-specific aminopeptidase of PA14 [25], we excluded dipeptides containing N- and C-terminal arginine residues.

### Multiple sequence alignment and phylogenetic analysis

Protein sequence data were obtained from NCBI and the Pseudomonas Genome Database [77]. Putative dipeptide ABC transporter proteins from *P. aeruginosa* PA14 were identified on basis of homology with already described transporters from other organisms found in the Transporter Classification Database [78] and respective BLASTP searches. A similar approach was used for the identification of substrate-binding protein homologs in PA14. Multiple sequence alignment was performed using ClustalO [79] and phylogenetic trees visualized by FigTree [80].

## Supporting Information

Figure S1 **Transmembrane domain analysis of the dipeptide transporter permease DppB and DppC.** The upper line indicates the predicted topology from TOPCONS [33] based on amino acid sequences. Red lines indicate an inner membrane orientation; blue lines indicate an outer membrane orientation; grey boxes indicate transmembrane helices spanning from the inside to the outside; white boxes indicate transmembrane helices spanning from the outside to the inside. Below the line is a graphical interpretation of the reliability of the prediction for each amino acid.
(TIF)

Figure S2 **Protein interaction model of the DppBCDF dipeptide transporter and its substrate-binding proteins from** ***P. aeruginosa*** **PAO1 predicted by the STRING database [81]** **.** The protein sequences from PAO1 are homologous to the following proteins from PA14: DppBCDF (PA4503–PA4506), DppA1 (PA4496), DppA2 (PA4497), DppA3 (PA4500), DppA4 (PA4502), and DppA5 (PA5317). Another ABC transporter system (PA2060–PA2061), homologous to PA14_37840, appears to interact with the dipeptide transporter network. Blue lines connecting the node spheres predict a physical or functional interaction between the proteins.
(TIF)

Figure S3 **Multiple sequence alignment of the amino acid sequences of DppA1, DppA2, DppA3, DppA4, and DppA5 from** ***P. aeruginosa*** **PA14 using Clustal Omega for analysis [79]** **and Jalview for data presentation [82]** **.** The percentage identity of each single residue is demonstrated by the blue color. The consensus sequence is shown below the alignment.
(TIF)

Figure S4 **Heatmap of di/tripeptide utilization by PA14, the** ***dppBCDF*** **mutant, and the SBP penta mutant.** The dipeptides shown in this figure were excluded from further analysis because they support the reduction of the tetrazolium dye also without detectable growth of the cells, or they are degraded by aminopeptidases secreted by PA14, or because the WT could not use these di/tripeptides as nitrogen source.
(TIF)

Figure S5 **Heatmap of di/tripeptide utilization by the SBP penta mutant and by strains of the penta mutant complemented with individual SBPs.** The dipeptides shown in this figure were excluded from further analysis because they support the reduction of the tetrazolium dye also without detectable growth of the cells, or they are degraded by aminopeptidases secreted by PA14, or because the WT could not use these di/tripeptides as nitrogen source.
(TIF)

Table S1 **Plasmids used in this study.**
(PDF)

Table S2 **Primers used in this study.**
(PDF)

Table S3 **Identity values of bacterial dipeptide permeases calculated by Clustal Omega.**
(PDF)

Table S4 **Identity values of dipeptide substrate-binding proteins from different Gram-negative bacteria calculated by Clustal Omega.**
(PDF)

Dataset S1 **Raw Quantitative PM Data.** Contains a complete listing of the contents of Biolog plates PM6 to PM8 utilized in this study. Data, representing the kinetic response as area under the curve for several replicates, are shown for the WT, the transporter mutant *dppBCDF*, the SBPs penta mutant, as well as the penta mutant complemented with plasmids carrying individual substrate-binding proteins encoded by the genes *dppA1* to *dppA5*. The area under the curve was used to calculate the signal values represented in the heat maps in Figure 4 as well as Figures S5 and S6.
(XLSX)

Dataset S2 **PM Signal Curves.** Each image provides the signal curves generated by the OmniLog software in each well of the 96-well plates PM6 to PM8 that constitute the nitrogen source utilization assay (see Dataset S1 for full listing of plate contents). The x-axis of each signal curve represents the 24-h time course and the y-axis the cellular growth response. The red colored areas represent the reference growth (e.g. wild type), the green colored areas the experiment growth (e.g. mutant), and the yellow colored areas the overlap between the reference and the experiment growth.
(PDF)

Dataset S3 **Raw Data for Data Analysis.** A database used for data analysis that contains the average values of the replicates for each single strain (see Dataset S1 for the raw data of each replicate). Additionally, the following tables were added: table ‘AA’ showing all possible amino acids and their specific side chain; table ‘chemical’ showing the chemical nature of the implemented nitrogen source, each di-/tripeptide split into single amino acids (AA1 to AA3), the core structural function group of each amino acid (Core_AA1 to Core_AA3), and the form of each amino acid. The tables are linked via the primary keys ‘Plate’ and ‘Well’ that represent each well of the 96-well PM plates. The table ‘AA’ is connected to table ‘chemical’ via each single amino acid.
(ACCDB)

## References

[1] Lister PD, Wolter DJ, Hanson ND (2009) Antibacterial-resistant *Pseudomonas aeruginosa*: clinical impact and complex regulation of chromosomally encoded resistance mechanisms. Clin Microbiol Rev 22: 582–610. 19822890 10.1128/CMR.00040-09 PMC2772362

[2] Bodey GP, Bolivar R, Fainstein V, Jadeja L (1983) Infections caused by *Pseudomonas aeruginosa* . Rev Infect Dis 5: 279–313. 6405475 10.1093/clinids/5.2.279

[3] Breidenstein EB, Bains M, Hancock RE (2012) Involvement of the lon protease in the SOS response triggered by ciprofloxacin in *Pseudomonas aeruginosa* PAO1. Antimicrob Agents Chemother 56: 2879–2887. 22450976 10.1128/AAC.06014-11 PMC3370746

[4] Bonomo RA, Szabo D (2006) Mechanisms of multidrug resistance in *Acinetobacter* species and *Pseudomonas aeruginosa* . Clin Infect Dis 43 Suppl 2 S49–56. 16894515 10.1086/504477

[5] Hancock RE, Speert DP (2000) Antibiotic resistance in *Pseudomonas aeruginosa*: mechanisms and impact on treatment. Drug Resist Updat 3: 247–255. 11498392 10.1054/drup.2000.0152

[6] Kelly-Wintenberg K, Montie TC (1994) Chemotaxis to oligopeptides by *Pseudomonas aeruginosa* . Appl Environ Microbiol 60: 363–367. 8117090 10.1128/aem.60.1.363-367.1994 PMC201315

[7] Stacey G, Koh S, Granger C, Becker JM (2002) Peptide transport in plants. Trends Plant Sci 7: 257–263. 12049922 10.1016/s1360-1385(02)02249-5

[8] Matthews D, Payne J (1980) Transmembrane transport of small peptides. In Current topics in membrane and transport. New York: Academic Press. Vol. 14, 331–425.

[9] Payne JW, Smith MW (1994) Peptide transport by micro-organisms. Adv Microb Physiol 36: 1–80. 7942312 10.1016/s0065-2911(08)60176-9

[10] Hiles ID, Gallagher MP, Jamieson DJ, Higgins CF (1987) Molecular characterization of the oligopeptide permease of *Salmonella typhimurium* . J Mol Biol 195: 125–142. 2821267 10.1016/0022-2836(87)90332-9

[11] Smith MW, Payne JW (1990) Simultaneous exploitation of different peptide permeases by combinations of synthetic peptide smugglins can lead to enhanced antibacterial activity. FEMS Microbiol Lett 58: 311–316. 2227366 10.1111/j.1574-6968.1990.tb13995.x

[12] Homann OR, Cai H, Becker JM, Lindquist SL (2005) Harnessing natural diversity to probe metabolic pathways. PLoS Genet 1: e80. 16429164 10.1371/journal.pgen.0010080 PMC1342634

[13] Ringrose PS (1980) Peptides as antimicrobial agents. In: Payne JW, editor. Microorganisms and nitrogen sources. Chichester, United Kingdom: John Wiley & Sons Ltd. pp. 641–657.

[14] Rubio-Aliaga I, Daniel H (2008) Peptide transporters and their roles in physiological processes and drug disposition. Xenobiotica 38: 1022–1042. 18668438 10.1080/00498250701875254

[15] Higgins CF (2001) ABC transporters: physiology, structure and mechanism – an overview. Res Microbiol 152: 205–210. 11421269 10.1016/s0923-2508(01)01193-7

[16] Abouhamad WN, Manson M, Gibson MM, Higgins CF (1991) Peptide transport and chemotaxis in *Escherichia coli* and *Salmonella typhimurium*: characterization of the dipeptide permease (Dpp) and the dipeptide-binding protein. Mol Microbiol 5: 1035–1047. 1956284 10.1111/j.1365-2958.1991.tb01876.x

[17] Weinberg MV, Maier RJ (2007) Peptide transport in *Helicobacter pylori*: roles of Dpp and Opp systems and evidence for additional peptide transporters. J Bacteriol 189: 3392–3402. 17322309 10.1128/JB.01636-06 PMC1855898

[18] Medrano MS, Ding Y, Wang XG, Lu P, Coburn J, et al (2007) Regulators of expression of the oligopeptide permease A proteins of *Borrelia burgdorferi* . J Bacteriol 189: 2653–2659. 17237172 10.1128/JB.01760-06 PMC1855802

[19] Wu TK, Wang YK, Chen YC, Feng JM, Liu YH, et al (2007) Identification of a *Vibrio furnissii* oligopeptide permease and characterization of its in vitro hemolytic activity. J Bacteriol 189: 8215–8223. 17873048 10.1128/JB.01039-07 PMC2168660

[20] Lamarque M, Aubel D, Piard JC, Gilbert C, Juillard V, et al (2011) The peptide transport system Opt is involved in both nutrition and environmental sensing during growth of *Lactococcus lactis* in milk. Microbiology 157: 1612–1619. 21393368 10.1099/mic.0.048173-0

[21] Abouhamad WN, Manson MD (1994) The dipeptide permease of *Escherichia coli* closely resembles other bacterial transport systems and shows growth-phase-dependent expression. Mol Microbiol 14: 1077–1092. 7536291 10.1111/j.1365-2958.1994.tb01340.x

[22] Doeven MK, van den Bogaart G, Krasnikov V, Poolman B (2008) Probing receptor-translocator interactions in the oligopeptide ABC transporter by fluorescence correlation spectroscopy. Biophys J 94: 3956–3965. 18212011 10.1529/biophysj.107.120964 PMC2367188

[23] Smith MW, Tyreman DR, Payne GM, Marshall NJ, Payne JW (1999) Substrate specificity of the periplasmic dipeptide-binding protein from *Escherichia coli*: experimental basis for the design of peptide prodrugs. Microbiology 145: 2891–2901. 10537211 10.1099/00221287-145-10-2891

[24] Tamber S, Ochs MM, Hancock RE (2006) Role of the novel OprD family of porins in nutrient uptake in *Pseudomonas aeruginosa* . J Bacteriol 188: 45–54. 16352820 10.1128/JB.188.1.45-54.2006 PMC1317591

[25] Luckett JC, Darch O, Watters C, Abuoun M, Wright V, et al (2012) A novel virulence strategy for *Pseudomonas aeruginosa* mediated by an autotransporter with arginine-specific aminopeptidase activity. PLoS Pathog 8: e1002854. 22927813 10.1371/journal.ppat.1002854 PMC3426542

[26] Miller RV, Becker JM (1978) Peptide utilization in *Pseudomonas aeruginosa*: evidence for membrane-associated peptidase. J Bacteriol 133: 165–171. 412832 10.1128/jb.133.1.165-171.1978 PMC221990

[27] Stover CK, Pham XQ, Erwin AL, Mizoguchi SD, Warrener P, et al (2000) Complete genome sequence of *Pseudomonas aeruginosa* PAO1, an opportunistic pathogen. Nature 406: 959–964. 10984043 10.1038/35023079

[28] Manson MD, Blank V, Brade G, Higgins CF (1986) Peptide chemotaxis in *E. coli* involves the Tap signal transducer and the dipeptide permease. Nature 321: 253–256. 3520334 10.1038/321253a0

[29] Elliott T (1993) Transport of 5-aminolevulinic acid by the dipeptide permease in *Salmonella typhimurium* . J Bacteriol 175: 325–331. 8380400 10.1128/jb.175.2.325-331.1993 PMC196145

[30] Verkamp E, Backman VM, Bjornsson JM, Soll D, Eggertsson G (1993) The periplasmic dipeptide permease system transports 5-aminolevulinic acid in *Escherichia coli* . J Bacteriol 175: 1452–1456. 8444807 10.1128/jb.175.5.1452-1456.1993 PMC193232

[31] Letoffe S, Delepelaire P, Wandersman C (2006) The housekeeping dipeptide permease is the *Escherichia coli* heme transporter and functions with two optional peptide binding proteins. Proc Natl Acad Sci U S A 103: 12891–12896. 16905647 10.1073/pnas.0605440103 PMC1568943

[32] Mistry A, Warren MS, Cusick JK, Karkhoff-Schweizer RR, Lomovskaya O, Schweizer HP (2013) High-level pacidamycin resistance in *Pseudomonas aeruginosa* is mediated by an Opp oligopeptide permease encoded by the *opp*-*fabI* operon. Antimicrob Agents Chemother 57: 5565–5571. 23979749 10.1128/AAC.01198-13 PMC3811240

[33] Bernsel A, Viklund H, Hennerdal A, Elofsson A (2009) TOPCONS: consensus prediction of membrane protein topology. Nucleic Acids Res 37: W465–468. 19429891 10.1093/nar/gkp363 PMC2703981

[34] de Jong A, Pietersma H, Cordes M, Kuipers OP, Kok J (2012) PePPER: a webserver for prediction of prokaryote promoter elements and regulons. BMC Genomics 13: 299. 22747501 10.1186/1471-2164-13-299 PMC3472324

[35] Wurtzel O, Yoder-Himes DR, Han K, Dandekar AA, Edelheit S, et al (2012) The single-nucleotide resolution transcriptome of *Pseudomonas aeruginosa* grown in body temperature. PLoS Pathog 8: e1002945. 23028334 10.1371/journal.ppat.1002945 PMC3460626

[36] Kiely PD, O'Callaghan J, Abbas A, O'Gara F (2008) Genetic analysis of genes involved in dipeptide metabolism and cytotoxicity in *Pseudomonas aeruginosa* PAO1. Microbiology 154: 2209–2218. 18667554 10.1099/mic.0.2007/015032-0

[37] Staskawicz BJ, Panopoulos NJ (1980) Phaseolotoxin transport in *Escherichia coli* and *Salmonella typhimurium* via the oligopeptide permease. J Bacteriol 142: 474–479. 6991475 10.1128/jb.142.2.474-479.1980 PMC294006

[38] Fuerst JA, Sagulenko E (2010) Protein uptake by bacteria: An endocytosis-like process in the planctomycete *Gemmata obscuriglobus* . Commun Integr Biol 3: 572–575. 21331243 10.4161/cib.3.6.13061 PMC3038067

[39] Kessler E, Safrin M, Olson JC, Ohman DE (1993) Secreted LasA of *Pseudomonas aeruginosa* is a staphylolytic protease. J Biol Chem 268: 7503–7508. 8463280

[40] Morihara K (1995) Pseudolysin and other pathogen endopeptidases of thermolysin family. Methods Enzymol 248: 242–253. 7674924 10.1016/0076-6879(95)48017-x

[41] Bardoel BW, van Kessel KP, van Strijp JA, Milder FJ (2012) Inhibition of *Pseudomonas aeruginosa* virulence: characterization of the AprA-AprI interface and species selectivity. J Mol Biol 415: 573–583. 22154939 10.1016/j.jmb.2011.11.039

[42] Engel LS, Hill JM, Caballero AR, Green LC, O'Callaghan RJ (1998) Protease IV, a unique extracellular protease and virulence factor from *Pseudomonas aeruginosa* . J Biol Chem 273: 16792–16797. 9642237 10.1074/jbc.273.27.16792

[43] Wilderman PJ, Vasil AI, Johnson Z, Wilson MJ, Cunliffe HE, et al (2001) Characterization of an endoprotease (PrpL) encoded by a PvdS-regulated gene in *Pseudomonas aeruginosa* . Infect Immun 69: 5385–5394. 11500408 10.1128/IAI.69.9.5385-5394.2001 PMC98648

[44] Cahan R, Axelrad I, Safrin M, Ohman DE, Kessler E (2001) A secreted aminopeptidase of *Pseudomonas aeruginosa*. Identification, primary structure, and relationship to other aminopeptidases. J Biol Chem 276: 43645–43652. 11533066 10.1074/jbc.M106950200

[45] Marquart ME, Caballero AR, Chomnawang M, Thibodeaux BA, Twining SS, et al (2005) Identification of a novel secreted protease from *Pseudomonas aeruginosa* that causes corneal erosions. Invest Ophthalmol Vis Sci 46: 3761–3768. 16186360 10.1167/iovs.04-1483

[46] Hulen C, Le Goffic F (1988) Peptidase N and alanyl-peptide transport in *Pseudomonas aeruginosa* . FEMS Microbiol Lett 49: 167–172.

[47] Guyer CA, Morgan DG, Staros JV (1986) Binding specificity of the periplasmic oligopeptide-binding protein from *Escherichia coli* . J Bacteriol 168: 775–779. 3536860 10.1128/jb.168.2.775-779.1986 PMC213550

[48] Letoffe S, Delepelaire P, Wandersman C (2008) Functional differences between heme permeases: *Serratia marcescens* HemTUV permease exhibits a narrower substrate specificity (restricted to heme) than the *Escherichia coli* DppABCDF peptide-heme permease. J Bacteriol 190: 1866–1870. 18178744 10.1128/JB.01636-07 PMC2258887

[49] Tam R, Saier MH Jr (1993) Structural, functional, and evolutionary relationships among extracellular solute-binding receptors of bacteria. Microbiol Rev 57: 320–346. 8336670 10.1128/mr.57.2.320-346.1993 PMC372912

[50] Cai H, Hauser M, Naider F, Becker JM (2007) Differential regulation and substrate preferences in two peptide transporters of *Saccharomyces cerevisiae* . Eukaryot Cell 6: 1805–1813. 17693598 10.1128/EC.00257-06 PMC2043388

[51] Perry D, Gilvarg C (1984) Spectrophotometric determination of affinities of peptides for their transport systems in *Escherichia coli* . J Bacteriol 160: 943–948. 6389518 10.1128/jb.160.3.943-948.1984 PMC215800

[52] Nickitenko AV, Trakhanov S, Quiocho FA (1995) 2 Å resolution structure of DppA, a periplasmic dipeptide transport/chemosensory receptor. Biochemistry 34: 16585–16595. 8527431 10.1021/bi00051a006

[53] Dunten P, Mowbray SL (1995) Crystal structure of the dipeptide binding protein from *Escherichia coli* involved in active transport and chemotaxis. Protein Sci 4: 2327–2334. 8563629 10.1002/pro.5560041110 PMC2143009

[54] Lin B, Short SA, Eskildsen M, Klempner MS, Hu LT (2001) Functional testing of putative oligopeptide permease (Opp) proteins of *Borrelia burgdorferi*: a complementation model in *opp* ^−^ *Escherichia coli* . Biochim Biophys Acta 1499: 222–231. 11341969 10.1016/s0167-4889(00)00121-x

[55] Wang XG, Kidder JM, Scagliotti JP, Klempner MS, Noring R, et al (2004) Analysis of differences in the functional properties of the substrate binding proteins of the *Borrelia burgdorferi* oligopeptide permease (Opp) operon. J Bacteriol 186: 51–60. 14679224 10.1128/JB.186.1.51-60.2004 PMC365673

[56] Shuman HA (1982) The maltose-maltodextrin transport system of *Escherichia coli* . Ann Microbiol (Paris) 133A: 153–159. 7041738

[57] Peltoniemi K, Vesanto E, Palva A (2002) Genetic characterization of an oligopeptide transport system from *Lactobacillus delbrueckii* subsp. *bulgaricus* . Arch Microbiol 177: 457–467. 12029391 10.1007/s00203-002-0411-9

[58] Wang XG, Lin B, Kidder JM, Telford S, Hu LT (2002) Effects of environmental changes on expression of the oligopeptide permease (*opp*) genes of *Borrelia burgdorferi* . J Bacteriol 184: 6198–6206. 12399490 10.1128/JB.184.22.6198-6206.2002 PMC151964

[59] van der Heide T, Poolman B (2002) ABC transporters: one, two or four extracytoplasmic substrate-binding sites? EMBO Rep 3: 938–943. 12370206 10.1093/embo-reports/kvf201 PMC1307614

[60] Salunkhe P, Smart CH, Morgan JA, Panagea S, Walshaw MJ, et al (2005) A cystic fibrosis epidemic strain of *Pseudomonas aeruginosa* displays enhanced virulence and antimicrobial resistance. J Bacteriol 187: 4908–4920. 15995206 10.1128/JB.187.14.4908-4920.2005 PMC1169510

[61] Ochsner UA, Wilderman PJ, Vasil AI, Vasil ML (2002) GeneChip expression analysis of the iron starvation response in *Pseudomonas aeruginosa*: identification of novel pyoverdine biosynthesis genes. Mol Microbiol 45: 1277–1287. 12207696 10.1046/j.1365-2958.2002.03084.x

[62] Bono JL, Tilly K, Stevenson B, Hogan D, Rosa P (1998) Oligopeptide permease in *Borrelia burgdorferi*: putative peptide-binding components encoded by both chromosomal and plasmid loci. Microbiology 144: 1033–1044. 9579077 10.1099/00221287-144-4-1033

[63] Torchilin V (2008) Intracellular delivery of protein and peptide therapeutics. Drug Discov Today Technol 5: e95–e103. 24981097 10.1016/j.ddtec.2009.01.002

[64] Bender CL, Alarcón-Chaidez F, Gross DC (1999) *Pseudomonas syringae* phytotoxins: mode of action, regulation, and biosynthesis by peptide and polyketide synthetases. Microbiol Mol Biol Rev 63: 266–292. 10357851 10.1128/mmbr.63.2.266-292.1999 PMC98966

[65] Zumaquero A, Macho AP, Rufian JS, Beuzon CR (2010) Analysis of the role of the type III effector inventory of *Pseudomonas syringae* pv. phaseolicola 1448a in interaction with the plant. J Bacteriol 192: 4474–4488. 20601478 10.1128/JB.00260-10 PMC2937392

[66] Schweizer HP, Hoang TT (1995) An improved system for gene replacement and *xylE* fusion analysis in *Pseudomonas aeruginosa* . Gene 158: 15–22. 7789804 10.1016/0378-1119(95)00055-b

[67] Hoang TT, Karkhoff-Schweizer RR, Kutchma AJ, Schweizer HP (1998) A broad-host-range Flp-*FRT* recombination system for site-specific excision of chromosomally-located DNA sequences: application for isolation of unmarked *Pseudomonas aeruginosa* mutants. Gene 212: 77–86. 9661666 10.1016/s0378-1119(98)00130-9

[68] Cherepanov PP, Wackernagel W (1995) Gene disruption in *Escherichia coli*: Tc^R^ and Km^R^ cassettes with the option of Flp-catalyzed excision of the antibiotic-resistance determinant. Gene 158: 9–14. 7789817 10.1016/0378-1119(95)00193-a

[69] Kovach ME, Elzer PH, Hill DS, Robertson GT, Farris MA, et al (1995) Four new derivatives of the broad-host-range cloning vector pBBR1MCS, carrying different antibiotic-resistance cassettes. Gene 166: 175–176. 8529885 10.1016/0378-1119(95)00584-1

[70] Hobert O (2002) PCR fusion-based approach to create reporter gene constructs for expression analysis in transgenic *C. elegans* . BioTechniques 32: 728–730. 11962590 10.2144/02324bm01

[71] Schenk A, Weingart H, Ullrich MS (2008) Extraction of high-quality bacterial RNA from infected leaf tissue for bacterial *in planta* gene expression analysis by multiplexed fluorescent Northern hybridization. Mol Plant Pathol 9: 227–235. 18705854 10.1111/j.1364-3703.2007.00452.x PMC6640379

[72] Köhler T, Ouertatani-Sakouhi H, Cosson P, van Delden C (2014) QsrO a novel regulator of quorum-sensing and virulence in *Pseudomonas aeruginosa* . PloS one 9: e87814. 24551066 10.1371/journal.pone.0087814 PMC3923755

[73] Arndt H, Henning C, Völksch B, Fritsche W (1989) Beziehung zwischen Virulenz und Phaseolotoxinbildungsvermögen bei verschiedenen *Pseudomonas syringae* pv. *phaseolicola*-Stämmen. Arch Phytopathol: 247–257.

[74] Wensing A, Braun SD, Büttner P, Expert D, Völksch B, et al (2010) Impact of siderophore production by *Pseudomonas syringae* pv. syringae 22d/93 on epiphytic fitness and biocontrol activity against *Pseudomonas syringae* pv. glycinea 1a/96. Appl Environ Microbiol 76: 2704–2711. 20208028 10.1128/AEM.02979-09 PMC2863448

[75] Bochner BR, Gadzinski P, Panomitros E (2001) Phenotype microarrays for high-throughput phenotypic testing and assay of gene function. Genome Res 11: 1246–1255. 11435407 10.1101/gr.186501 PMC311101

[76] Microsoft (2007) Microsoft Access 2007. Redmond, Washington: Computer Software.

[77] Winsor GL, Lam DK, Fleming L, Lo R, Whiteside MD, et al (2011) Pseudomonas Genome Database: improved comparative analysis and population genomics capability for *Pseudomonas* genomes. Nucleic Acids Res 39: D596–600. 20929876 10.1093/nar/gkq869 PMC3013766

[78] Saier MH Jr, Reddy VS, Tamang DG, Vastermark A (2014) The transporter classification database. Nucleic Acids Res 42: D251–258. 24225317 10.1093/nar/gkt1097 PMC3964967

[79] Sievers F, Wilm A, Dineen D, Gibson TJ, Karplus K, et al (2011) Fast, scalable generation of high-quality protein multiple sequence alignments using Clustal Omega. Mol Syst Biol 7: 539. 21988835 10.1038/msb.2011.75 PMC3261699

[80] Gonnet GH, Hallett MT, Korostensky C, Bernardin L (2000) Darwin v. 2.0: an interpreted computer language for the biosciences. Bioinformatics 16: 101–103. 10842729 10.1093/bioinformatics/16.2.101

[81] Szklarczyk D, Franceschini A, Kuhn M, Simonovic M, Roth A, et al (2011) The STRING database in 2011: functional interaction networks of proteins, globally integrated and scored. Nucleic Acids Res 39: D561–568. 21045058 10.1093/nar/gkq973 PMC3013807

[82] Waterhouse AM, Procter JB, Martin DM, Clamp M, Barton GJ (2009) Jalview Version 2 – a multiple sequence alignment editor and analysis workbench. Bioinformatics 25: 1189–1191. 19151095 10.1093/bioinformatics/btp033 PMC2672624

[83] Thoma S, Schobert M (2009) An improved *Escherichia coli* donor strain for diparental mating. FEMS Microbiol Lett 294: 127–132. 19431232 10.1111/j.1574-6968.2009.01556.x

[84] He J, Baldini RL, Deziel E, Saucier M, Zhang Q, et al (2004) The broad host range pathogen *Pseudomonas aeruginosa* strain PA14 carries two pathogenicity islands harboring plant and animal virulence genes. Proc Natl Acad Sci U S A 101: 2530–2535. 14983043 10.1073/pnas.0304622101 PMC356984

