# Supplementary figures and images for: High-Throughput Screening of Dipeptide Utilization Mediated by the ABC Transporter DppBCDF and Its Substrate-Binding Proteins DppA1-A5 in Pseudomonas aeruginosa
Source: PLoS One. 2014 Oct 22;9(10):e111311. doi: 10.1371/journal.pone.0111311 (PMC4206461; doi:10.1371/journal.pone.0111311)

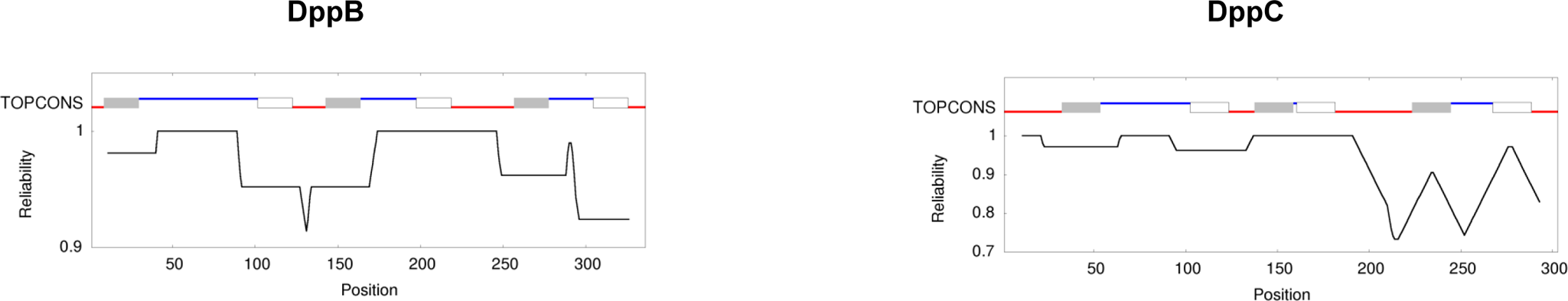

Supplement: Figure S1 — Transmembrane domain analysis of the dipeptide transporter permease DppB and DppC. The upper line indicates the predicted topology from TOPCONS [33] based on amino acid sequences. Red lines indicate an inner membrane orientation; blue lines indicate an outer membrane orientation; grey boxes indicate transmembrane helices spanning from the inside to the outside; white boxes indicate transmembrane helices spanning from the outside to the inside. Below the line is a graphical interpretation of the reliability of the prediction for each amino acid. (TIF) [file pone.0111311.s001.tif]

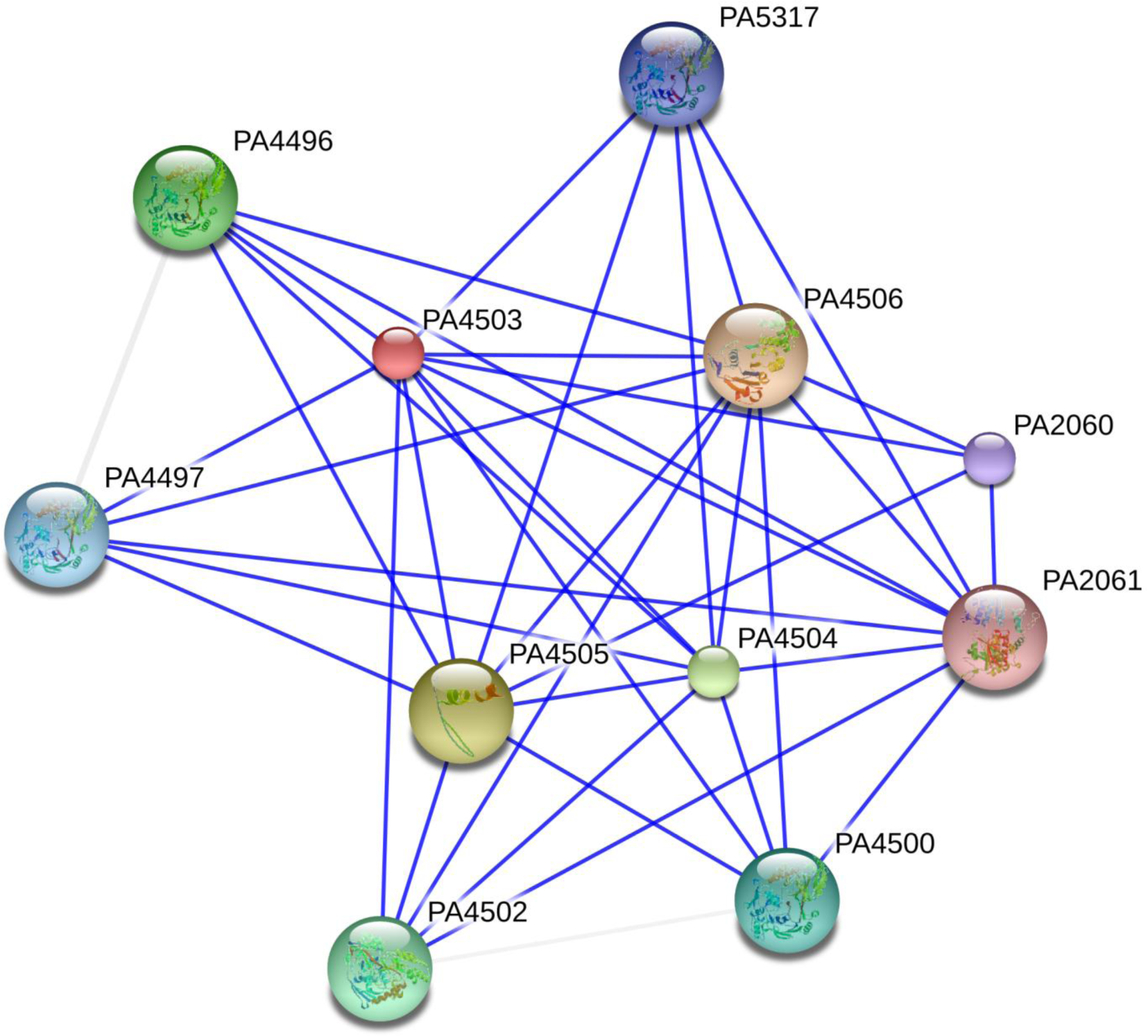

Supplement: Figure S2 — Protein interaction model of the DppBCDF dipeptide transporter and its substrate-binding proteins from P. aeruginosa PAO1 predicted by the STRING database [81] . The protein sequences from PAO1 are homologous to the following proteins from PA14: DppBCDF (PA4503–PA4506), DppA1 (PA4496), DppA2 (PA4497), DppA3 (PA4500), DppA4 (PA4502), and DppA5 (PA5317). Another ABC transporter system (PA2060–PA2061), homologous to PA14_37840, appears to interact with the dipeptide transporter network. Blue lines connecting the node spheres predict a physical or functional interaction between the proteins. (TIF) [file pone.0111311.s002.tif]

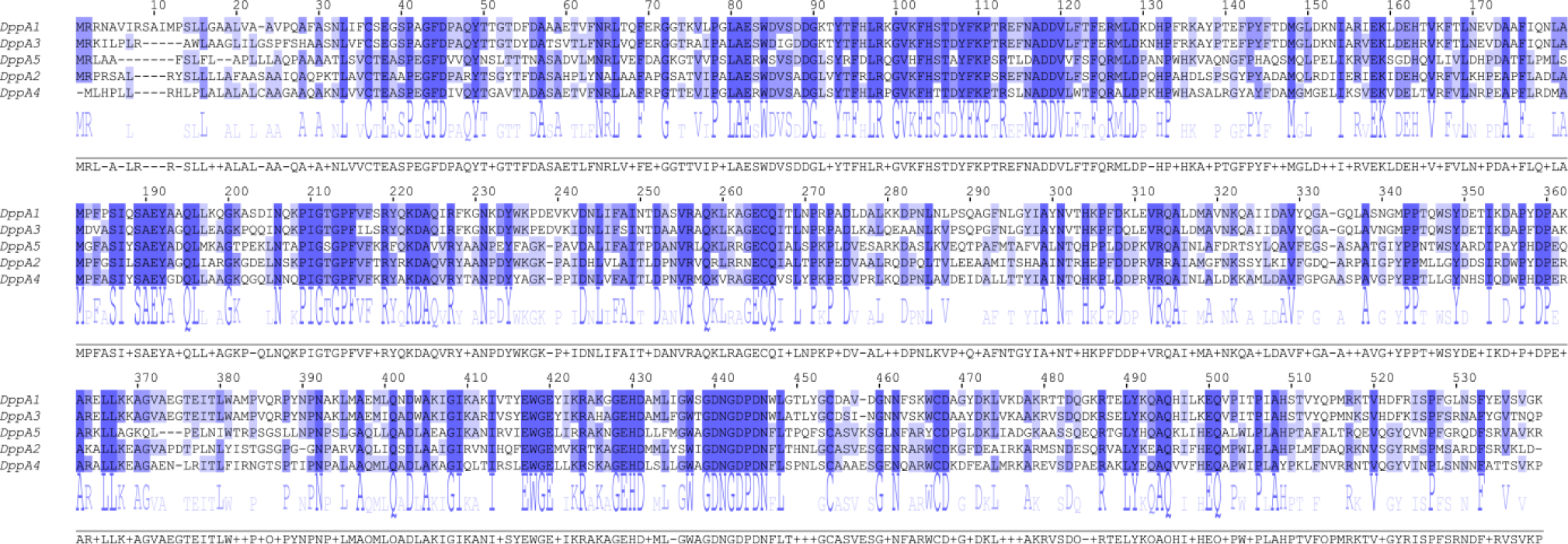

Supplement: Figure S3 — Multiple sequence alignment of the amino acid sequences of DppA1, DppA2, DppA3, DppA4, and DppA5 from P. aeruginosa PA14 using Clustal Omega for analysis [79] and Jalview for data presentation [82] . The percentage identity of each single residue is demonstrated by the blue color. The consensus sequence is shown below the alignment. (TIF) [file pone.0111311.s003.tif]

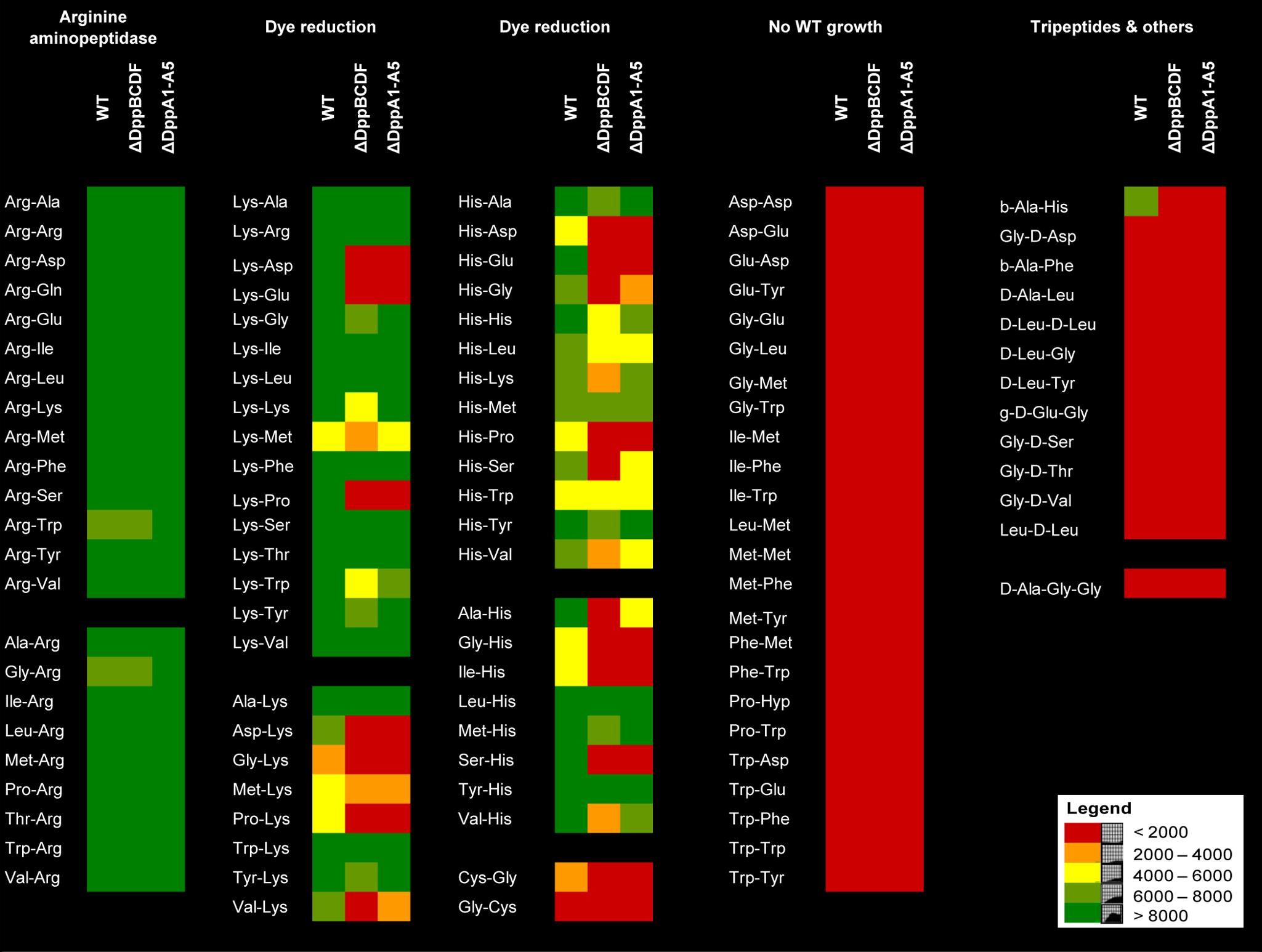

Supplement: Figure S4 — Heatmap of di/tripeptide utilization by PA14, the dppBCDF mutant, and the SBP penta mutant. The dipeptides shown in this figure were excluded from further analysis because they support the reduction of the tetrazolium dye also without detectable growth of the cells, or they are degraded by aminopeptidases secreted by PA14, or because the WT could not use these di/tripeptides as nitrogen source. (TIF) [file pone.0111311.s004.tif]

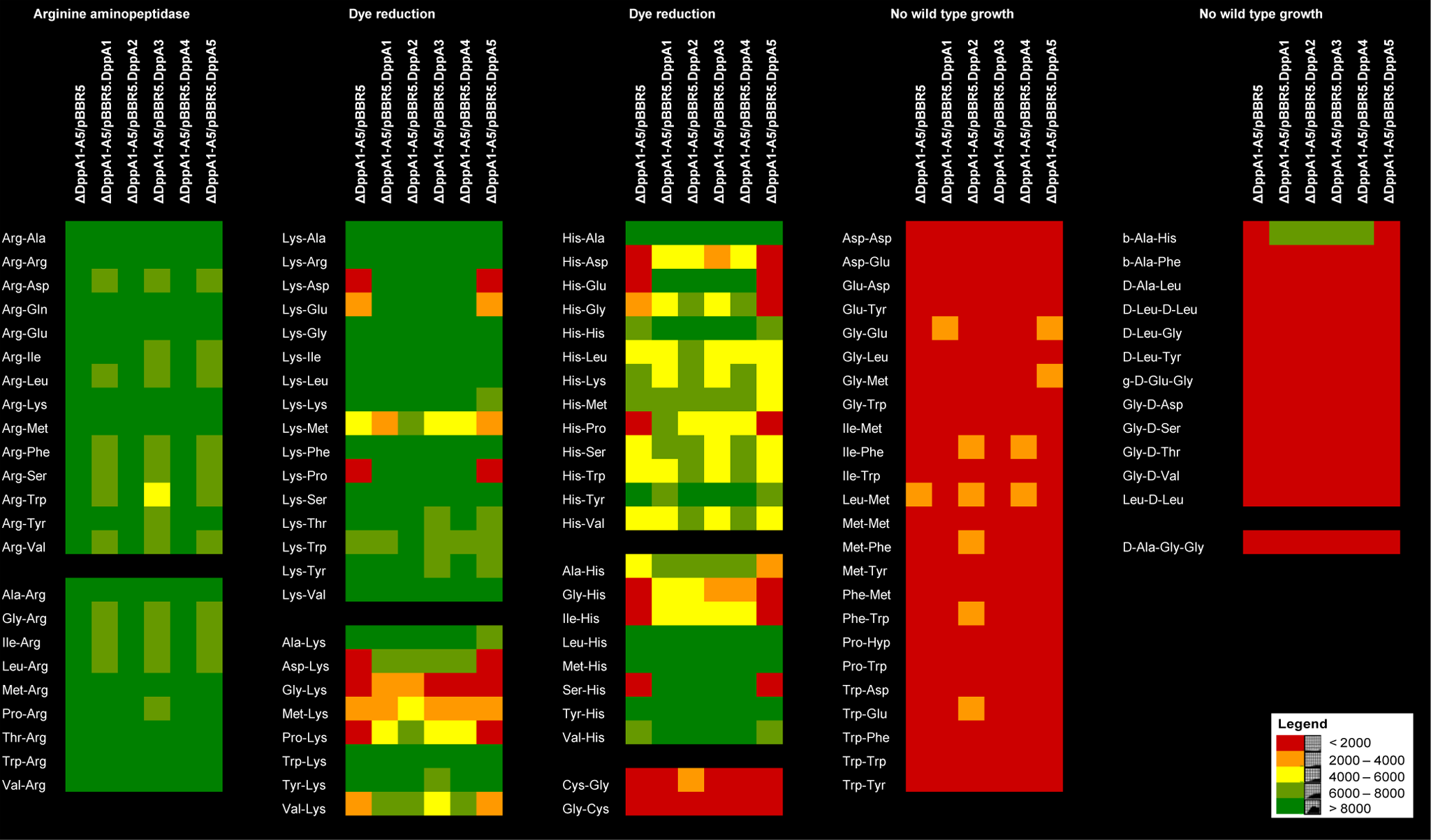

Supplement: Figure S5 — Heatmap of di/tripeptide utilization by the SBP penta mutant and by strains of the penta mutant complemented with individual SBPs. The dipeptides shown in this figure were excluded from further analysis because they support the reduction of the tetrazolium dye also without detectable growth of the cells, or they are degraded by aminopeptidases secreted by PA14, or because the WT could not use these di/tripeptides as nitrogen source. (TIF) [file pone.0111311.s005.tif]
