# Supplementary material for: High-Throughput Screening of Dipeptide Utilization Mediated by the ABC Transporter DppBCDF and Its Substrate-Binding Proteins DppA1-A5 in Pseudomonas aeruginosa
Source: PLoS One. 2014 Oct 22;9(10):e111311. doi: 10.1371/journal.pone.0111311 (PMC4206461; doi:10.1371/journal.pone.0111311)
Supplement: Table S1 — Plasmids used in this study. (PDF) [file pone.0111311.s006.pdf]

Table S1. Plasmids used in this study.

Plasmid	Relevant characteristics[a]	Reference or source
pGEM-T Easy	Ap^r, cloning vector	Promega
pPS858-Eco	Ap^r, Gm^r, source of Gm^r-GFP-*FRT* cassette	[67]
pEX18Ap	Ap^r, gene replacement vector	[67]
pEX18Ap.ABC58-ko.Gm-GFP	Ap^r, Gm^r, contains a 2.8-kb fusion fragment of PA14_58420, Gm^r-GFP-*FRT* cassette, and PA14_58500	This study
pEX18Gm	Gm^r, gene replacement vector	[67]
pEX18Gm.SBP58350-ko	Gm^r, contains a 1.1-kb fusion fragment of PA14_58350 and PA14_58360	This study
pEX18Gm.SBP58390-ko	Gm^r, contains a 1.5-kb fusion fragment of PA14_58390	This study
pEX18Gm.SBP58420-ko	Gm^r, contains a 1.2-kb fusion fragment of PA14_58420	This study
pEX18Gm.SBP70200-ko	Gm^r, contains a 1.4-kb fusion fragment of PA14_70200	This study
pBBR1MCS-5	Gm^r, broad-host-range cloning vector	[69]
pBBR5.DppA1	Gm^r, contains a 2.0-kb fragment carrying the SBP PA14_58350 including upstream promoter region in opposite orientation with respect to the *lac* promoter	This study
pBBR5.DppA2	Gm^r, contains a 1.7-kb fragment carrying the SBP PA14_58360 including upstream promoter region in opposite orientation with respect to the *lac* promoter	This study
pBBR5.DppA3	Gm^r, contains a 1.9-kb fragment carrying the SBP PA14_58390 including upstream promoter region in opposite orientation with respect to the *lac* promoter	This study
pBBR5.DppA4	Gm^r, contains a 1.8-kb fusion fragment carrying the SBP PA14_58420 including upstream promoter region in opposite orientation with respect to the *lac* promoter	This study
pBBR5.DppA5	Gm^r, contains a 2.0-kb fragment carrying the SBP PA14_70200 including upstream promoter region in opposite orientation with respect to the *lac* promoter	This study
pFLP2	Ap^r, source of FLP recombinase	[67]

[a] Antibiotic resistance: Ap^r, ampicillin resistance; Gm^r, gentamicin resistance
