# Supplementary material for: High-Throughput Screening of Dipeptide Utilization Mediated by the ABC Transporter DppBCDF and Its Substrate-Binding Proteins DppA1-A5 in Pseudomonas aeruginosa
Source: PLoS One. 2014 Oct 22;9(10):e111311. doi: 10.1371/journal.pone.0111311 (PMC4206461; doi:10.1371/journal.pone.0111311)
Supplement: Table S2 — Primers used in this study. (PDF) [file pone.0111311.s007.pdf]

Table S2. Primers used in this study.

Primer	Sequence (5' - 3')	Characteristic(s)
**ABC transporter DppBCDF knockout (PA14_58440 – PA14_58490)**		
58420-A1	TGCTGCAAGCAGACCTGG	used to clone *dppBCDF* knockout vector
58420-A2	CCCTATAGTGAGTCGGTACCGTTGGCGGGTAACTCCTC	used to clone *dppBCDF* knockout vector
58490-B1	GGTACCGACTCACTATAGGGGCAATTCCTCGGCTGAGC	used to clone *dppBCDF* knockout vector
58500-B2	TGCTGTTCCGTACCGCTC	used to clone *dppBCDF* knockout vector
58420_out1	ACCCTGCTCGGCTACAAC	Primer flanking *dppBCDF* knockout fragment (used to confirm Gm-GFP cassette insertion)
58500_out2	TTCCTGATTGCCCTGGCG	Primer flanking *dppBCDF* knockout fragment (used to confirm Gm-GFP cassette insertion)
**Substrate-binding protein knockouts**		
SBP-58350_fwd_Hind	CTAAGCTTGAAGATCACTGGCTGGCG	used to clone PA14_58350, PA14_58360 knockout vector
SBP-58350_rev	CCGCGTGCGGCGCAGCGAGGAGTGACCTCTGGTTATTTTGA	used to clone PA14_58350, PA14_58360 knockout vector
SBP-58360_fwd	TCAAAATAACCAGAGGTCACTCCTCGCTGCGCCGCACGCGG	used to clone PA14_58350, PA14_58360 knockout vector
SBP-58360_rev_EcoRI	GCGAATTCTGGGGGATGG	used to clone PA14_58350, PA14_58360 knockout vector
SBP-58350_out1	GAAACCACCAGCGTCCTC	Primer flanking PA14_58350, PA14_58360 knockout fragment
SBP-58350_out2	TGAAGGATGGCGACATGG	Primer flanking PA14_58350, PA14_58360 knockout fragment
SBP-58390_fwd_Hind	ATAAGCTTCCTCTCCCAGGCG	used to clone PA14_58390 knockout vector
SBP-58390_rev	GACCGGGGCGGCGTCGGCAGCAAACCTCATTTTTTTTTTC	used to clone PA14_58390 knockout vector
SBP-58390_fwd	GAAAAAGAAAAAATGAGGTTTGCTGCCGACGCCGCCCCGGTC	used to clone PA14_58390 knockout vector
SBP-58390_rev_EcoRI	TGAATTCGGTGTTCGAGGC	used to clone PA14_58390 knockout vector
SBP-58390_out1	TGACCGTAGACCGCATCG	Primer flanking PA14_58390 knockout fragment
SBP-58390_out2	TTGTCCAGCTCCTCGCTG	Primer flanking PA14_58390 knockout fragment
SBP-58420_fwd_Hind	ATAAGCTTCCGCAAGGTCAGC	used to clone PA14_58420 knockout vector
SBP-58420_rev	GGCTGTGGTCCTTGTTCTTGGGGTCCTCCTCGTGGGC	used to clone PA14_58420 knockout vector
SBP-58420_fwd	GCCCACGAGGAGGACCCCAAGAACAAGGACCACAGCC	used to clone PA14_58420 knockout vector
SBP-58420_rev_EcoRI	AGGAATTCGTGCCAGACG	used to clone PA14_58420 knockout vector
SBP-58420_out1	TTCCTGGTCAACACTCCG	Primer flanking PA14_58420 knockout fragment
SBP-58420_out2	ATGGTCGAACAGCGAACC	Primer flanking PA14_58420 knockout fragment
SBP-70200_fwd_Hind	TCAAGCTTGTTCTTGTCGGTGG	used to clone PA14_70200 knockout vector
SBP-70200_rev	GTTCTCCCGGGCGTGGCCTGACGGGCTCCATTCGAACG	used to clone PA14_70200 knockout vector
SBP-70200_fwd	CGTTCGAATGGAGCCCGTCAGGCCACGCCCGGGAGAAC	used to clone PA14_70200 knockout vector
SBP-70200_rev_EcoRI	TAGAATTCGATACTGGAGAGCCC	used to clone PA14_70200 knockout vector
SBP-70200_out1	TTCGCAAGGTGCCGGATC	Primer flanking PA14_70200 knockout fragment
SBP-70200_out2	TCGTTCTGCGGCCATCTC	Primer flanking PA14_70200 knockout fragment
Substrate binding proteins overexpression		
SBP+Pro_58350_fwd(SacI)	GAGCTCATGAAGGCCGACGCCAGCTT	used to clone PA14_58350 overexpression vector
SBP+Pro_58350_rev(ApaI)	GGGCCCTACTTGCCGACGCTGACCT	used to clone PA14_58350 overexpression vector
SBP+Pro_58360_fwd(SacI)	GAGCTCAAGTAGCTTCGCCTGCCAGC	used to clone PA14_58360 overexpression vector
SBP+Pro_58360_rev(ApaI)	GGGCCCGATCAGTCCAGCTTCACCCG	used to clone PA14_58360 overexpression vector
SBP+Pro_58390_fwd(SacI)	GAGCTCTGTTCGACGACCATCCGACG	used to clone PA14_58390 overexpression vector
SBP+Pro_58390_rev(ApaI)	GGGCCCTCAGGGCTGGTTGGTTACGC	used to clone PA14_58390 overexpression vector
SBP+Pro_58420_fwd(XbaI)	TCTAGATAACCAACCAGCCCTGATG	used to clone PA14_58420 overexpression vector
SBP+Pro_58420_rev	GAGCAACGGATGGAGCATGGCTGATTGCTCCCTTTATTGTTG	used to clone PA14_58420 overexpression vector
SBP-Pro_58420_fwd	CAACAATAAAGGGAGCAATCAGCCATGCTCCATCCGTTGCTC	used to clone PA14_58420 overexpression vector
SBP-Pro_58420_rev(ApaI)	GGGCCCTCTAGGGCTTCACCGAGGTG	used to clone PA14_58420 overexpression vector
SBP+Pro_70200_fwd(SacI)	GAGCTCGGCGGGTTTTGTTGTGTGCG	used to clone PA14_70200 overexpression vector
SBP+Pro_70200_rev(ApaI)	GGGCCCTTCAGCGTTTCACGGCGACC	used to clone PA14_70200 overexpression vector

Primer	Sequence (5' - 3')	Characteristic(s)
Quantitative RT-PCR		
SBP58350_RT_fwd	GTAGAAACGCCGTCATCCG	
SBP58350_RT_rev	GCGGTTGAAGACGGTTTCC	
SBP58360_RT_fwd	TTCGATCCGGCCCGCTATA	
SBP58360_RT_rev	TGGAATTTCACGCCCTGGC	
SBP58390_RT_fwd	GCAACCTGGTGTTCTGCTC	
SBP58390_RT_rev	TGTAGGTCTTGCCGTCGTC	
SBP58420_RT_fwd	CGGAAGGCTTCGACATCGT	
SBP58420_RT_rev	GGCGCAGGTGGAAGGTATA	
SBP70200_RT_fwd	AGTGTGTGTACCGAGGCCA	
SBP70200_RT_rev	AAGCGATAGCTCAGGCCGT	
rpsL-F	GCAAGCGCATGGTCGACAAGA	
rpsL-R	CGCTGTGCTCTTGCAGGTTGTGA	
