# Supplementary material for: High-Throughput Screening of Dipeptide Utilization Mediated by the ABC Transporter DppBCDF and Its Substrate-Binding Proteins DppA1-A5 in Pseudomonas aeruginosa
Source: PLoS One. 2014 Oct 22;9(10):e111311. doi: 10.1371/journal.pone.0111311 (PMC4206461; doi:10.1371/journal.pone.0111311)
Supplement: Table S3 — Identity values of bacterial dipeptide permeases calculated by Clustal Omega. (PDF) [file pone.0111311.s008.pdf]

Table S3. Identity values of bacterial dipeptide permeases calculated by Clustal Omega.[a]

		A	B	C	D	E	F	G	H	I
**A**	TppB *Escherichia coli* (AAC74706)	**100**	14	17	19	17	19	15	18	18
**B**	NikB *Escherichia coli* (AAC76502)	14	**100**	38	39	40	37	36	39	37
**C**	AccD *Agrobacterium tumefaciens* (YP_001967616)	17	38	**100**	38	41	34	37	39	36
**D**	DdpB *Escherichia coli* (AAC74559)	19	39	38	**100**	44	46	42	45	44
**E**	GsiC *Escherichia coli* (AAC73918)	17	40	41	44	**100**	41	45	43	46
**F**	DppB *Haemophilus influenzae* (P45096)	19	37	34	46	41	**100**	**61**	**64**	**61**
**G**	DppB *Escherichia coli* (AAC76568)	15	36	37	42	45	**61**	**100**	**66**	**66**
**H**	DppB *Burkholderia pseudomallei* (YP_106877)	18	39	39	45	43	**64**	**66**	**100**	**74**
**I**	DppB *Pseudomonas aeruginosa* (ABJ13770)	18	37	36	44	46	**61**	**66**	**74**	**100**

[a] Bold face numbers indicate an identity of more than 60%.
