# Supplementary material for: High-Throughput Screening of Dipeptide Utilization Mediated by the ABC Transporter DppBCDF and Its Substrate-Binding Proteins DppA1-A5 in Pseudomonas aeruginosa
Source: PLoS One. 2014 Oct 22;9(10):e111311. doi: 10.1371/journal.pone.0111311 (PMC4206461; doi:10.1371/journal.pone.0111311)
Supplement: Table S4 — Identity values of dipeptide substrate-binding proteins from different Gram-negative bacteria calculated by Clustal Omega. (PDF) [file pone.0111311.s009.pdf]

Table S4. Identity values of dipeptide substrate-binding proteins from different Gram-negative bacteria calculated by Clustal Omega.[a]

		A	B	C	D	E	F	G	H	I	J	K	L	M	N
**A**	NikA *Escherichia coli* (AAB18451)	**100**	21	24	24	23	24	25	25	24	24	25	26	26	26
**B**	DppA5 *Pseudomonas aeruginosa* (YP_793791)	21	**100**	**64**	**64**	53	52	41	41	45	44	42	45	44	43
**C**	BDL_1606 *Burkholderia pseudomallei* (YP_008341763)	24	**64**	**100**	**99**	53	46	47	47	48	45	43	43	42	44
**D**	NikA *Burkholderia pseudomallei* (WP_004530979)	24	**64**	**99**	**100**	52	46	47	47	48	45	43	43	42	44
**E**	DppA4 *Pseudomonas aeruginosa* (YP_792847)	23	53	53	52	**100**	59	47	47	49	47	44	49	49	51
**F**	DppA2 *Pseudomonas aeruginosa* (YP_792842)	24	52	46	46	59	**100**	46	46	48	46	41	44	44	47
**G**	DppA *Burkholderia pseudomallei* (YP_106876)	25	41	47	47	47	46	**100**	**100**	**66**	**64**	45	55	55	51
**H**	DppA *Burkholderia pseudomallei* (WP_004534193)	25	41	47	47	47	46	**100**	**100**	**66**	**64**	45	55	55	51
**I**	DppA1 *Pseudomonas aeruginosa* (YP_792841)	24	45	48	48	49	48	**66**	**66**	**100**	**83**	46	51	51	54
**J**	DppA3 *Pseudomonas aeruginosa* (YP_792845)	24	44	45	45	47	46	**64**	**64**	**83**	**100**	46	51	51	52
**K**	HbpA *Haemophilus influenzae* (P33950)	25	42	43	43	44	41	45	45	46	46	**100**	53	53	49
**L**	DppA *Escherichia coli* (NP_418001)	26	45	43	43	49	44	55	55	51	51	53	**100**	**100**	54
**M**	DppA *Shigella flexneri* (NP_709323)	26	44	42	42	49	44	55	55	51	51	53	**100**	**100**	54
**N**	DppA *Brucella abortus* (WP_006088507)	26	43	44	44	51	47	51	51	54	52	49	54	54	**100**

[a] Bold face numbers indicate an identity of more than 60%.
