# Supplementary material for: High-Throughput Screening of Dipeptide Utilization Mediated by the ABC Transporter DppBCDF and Its Substrate-Binding Proteins DppA1-A5 in Pseudomonas aeruginosa
Source: PLoS One. 2014 Oct 22;9(10):e111311. doi: 10.1371/journal.pone.0111311 (PMC4206461; doi:10.1371/journal.pone.0111311)

Dataset 2

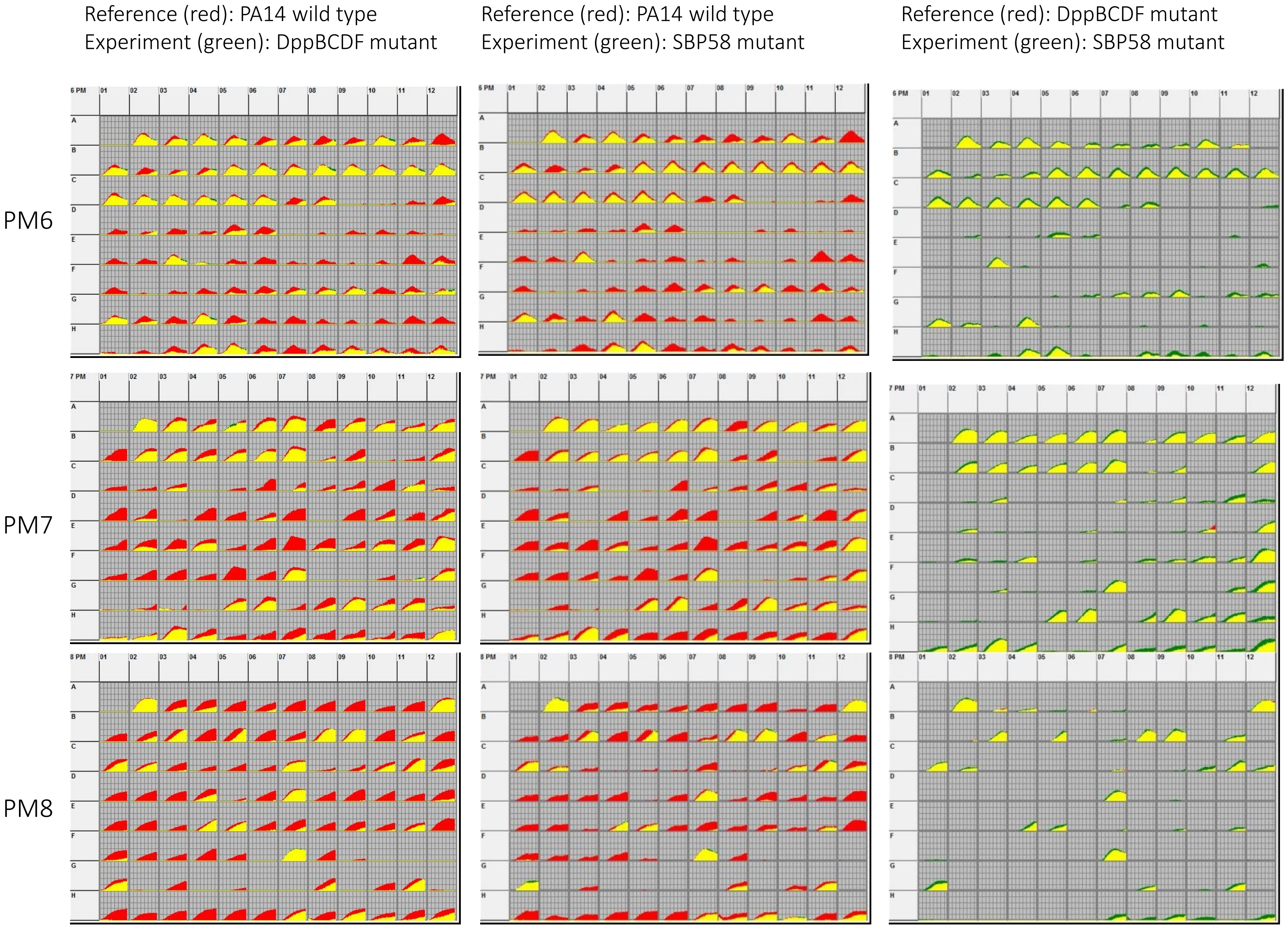
Reference (red): PA14 wild type
Experiment (green): DppBCDF mutant
Reference (red): PA14 wild type
Experiment (green): SBP58 mutant
Reference (red): DppBCDF mutant
Experiment (green): SBP58 mutant
PM6
PM7
PM8

Reference (red): ΔSBP58
Experiment (green):
ΔSBP58-pBBR5.58350

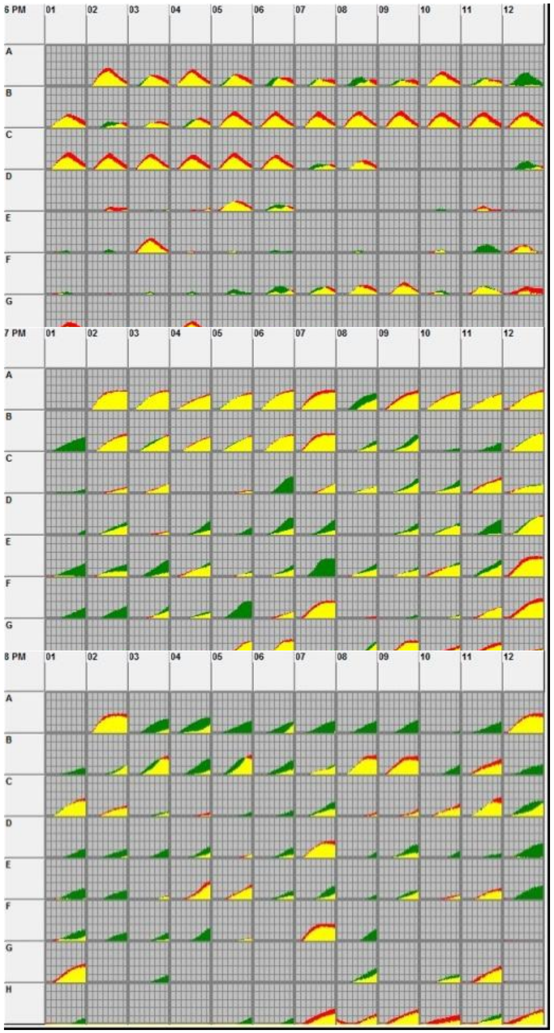

Reference (red): ΔSBP58
Experiment (green):
ΔSBP58-pBBR5.58360

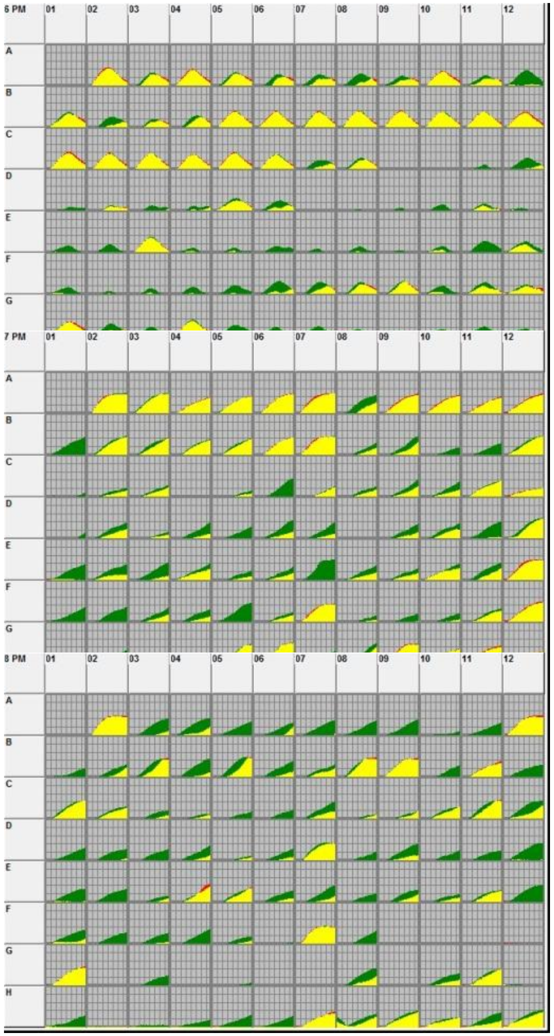

Reference (red): ΔSBP58
Experiment (green):
ΔSBP58-pBBR5.58390

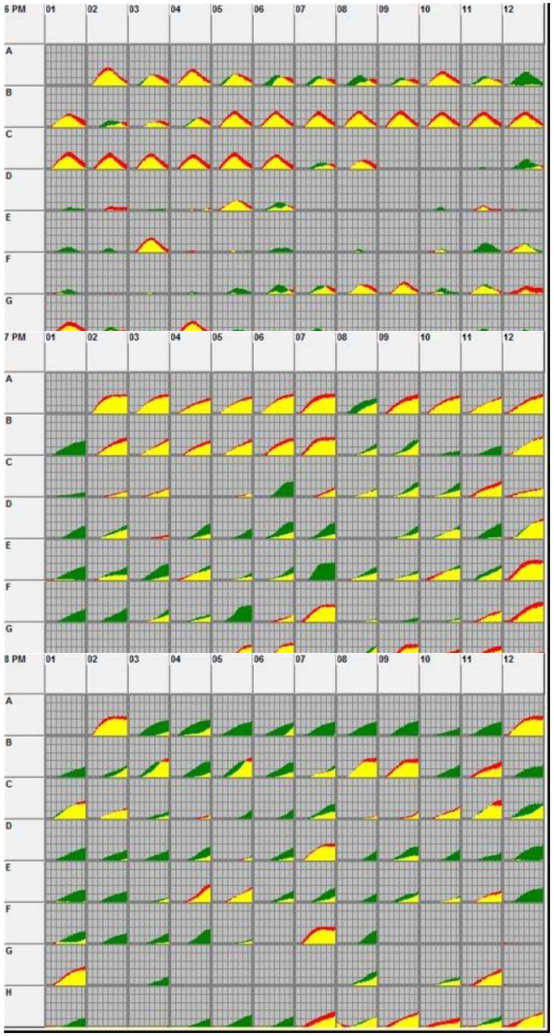

PM6

PM7

PM8

Reference (red): ΔSBP58
Experiment (green):
ΔSBP58-pBBR5.58420

PM6

PM7

PM8

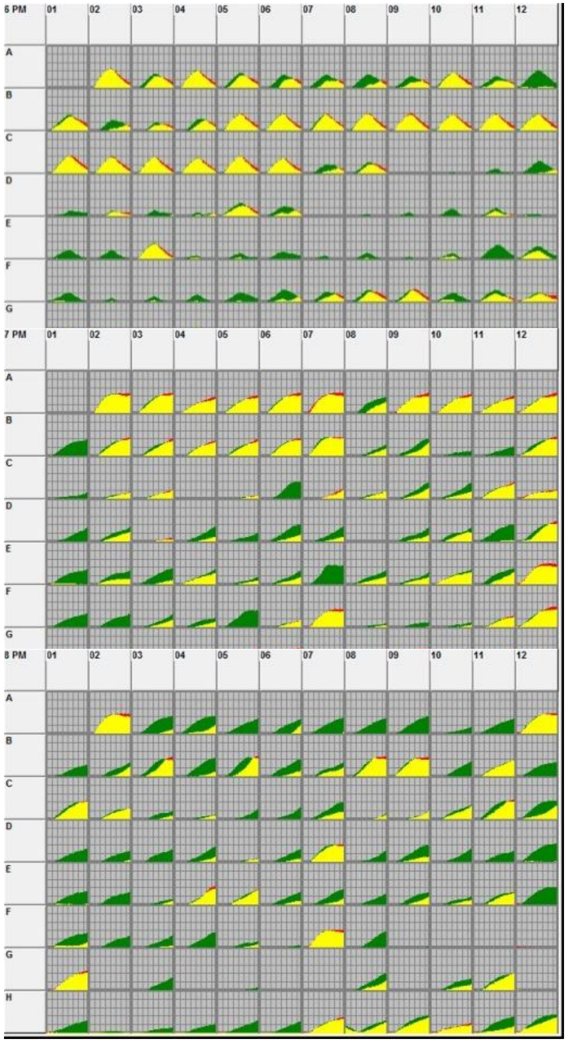

Reference (red): ΔSBP58
Experiment (green):
ΔSBP58-pBBR5.70200

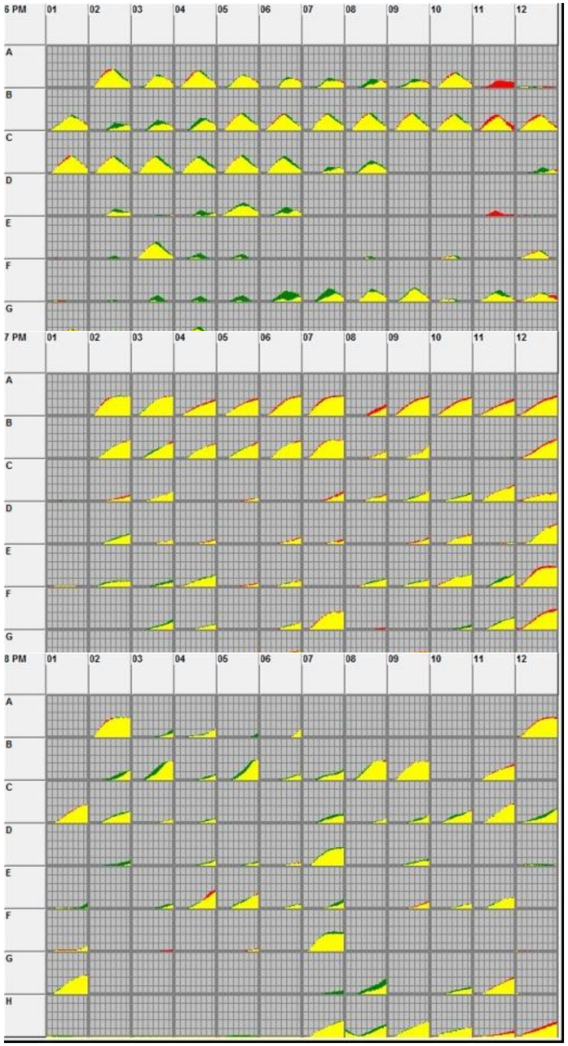

Supplement: Dataset S2 — PM Signal Curves. Each image provides the signal curves generated by the OmniLog software in each well of the 96-well plates PM6 to PM8 that constitute the nitrogen source utilization assay (see Dataset S1 for full listing of plate contents). The x-axis of each signal curve represents the 24-h time course and the y-axis the cellular growth response. The red colored areas represent the reference growth (e.g. wild type), the green colored areas the experiment growth (e.g. mutant), and the yellow colored areas the overlap between the reference and the experiment growth. (PDF) [file pone.0111311.s011.pdf]
